# Parallel Gene Expression Changes in Ventral Midbrain Dopamine and GABA Neurons during Normal Aging

**DOI:** 10.1523/ENEURO.0107-25.2025

**Published:** 2025-05-29

**Authors:** Ana Luiza Drumond-Bock, Harris E. Blankenship, Kevin D. Pham, Kelsey A. Carter, Willard M. Freeman, Michael J. Beckstead

**Affiliations:** ^1^Aging and Metabolism Research Program, Oklahoma Medical Research Foundation, Oklahoma City, Oklahoma 73104; ^2^Department of Biochemistry and Physiology, University of Oklahoma Health Sciences Center, Oklahoma City, Oklahoma 73104; ^3^Genes and Human Disease Research Program, Oklahoma Medical Research Foundation, Oklahoma City, Oklahoma 73104; ^4^Oklahoma City VA Medical Center, Oklahoma City, Oklahoma 73104

**Keywords:** mice, neuronal aging, serotonin, substantia nigra, translatomics, ventral tegmental area

## Abstract

The consequences of aging can vary dramatically between different brain regions and cell types. In the ventral midbrain, dopaminergic neurons develop physiological deficits with normal aging that likely convey susceptibility to neurodegeneration. While nearby GABAergic neurons are thought to be more resilient, decreased GABA signaling in other areas nonetheless correlates with age-related cognitive decline and the development of degenerative diseases. Here, we used two novel cell type-specific translating ribosome affinity purification models to elucidate the impact of healthy brain aging on the molecular profiles of dopamine and GABA neurons in the ventral midbrain. By analyzing differential gene expression from young adult (7-10 months) and old (21-24 months) mice, we detected commonalities in the aging process in both neuronal types, including increased inflammatory responses and upregulation of pro-survival pathways. Both cell types also showed downregulation of genes involved in synaptic connectivity and plasticity. Intriguingly, genes involved in serotonergic synthesis were upregulated with age in GABA neurons and not dopamine-releasing cells. In contrast, dopaminergic neurons showed alterations in genes connected with mitochondrial function and calcium signaling, which were markedly downregulated in male mice. Sex differences were detected in both neuron types, but in general were more prominent in dopamine neurons. Multiple sex effects correlated with the differential prevalence for neurodegenerative diseases such as Parkinson's and Alzheimer's seen in humans. In summary, these results provide insight into the connection between non-pathological aging and susceptibility to neurodegenerative diseases involving the ventral midbrain, and identify molecular phenotypes that could underlie homeostatic maintenance during normal aging.

## Significance Statement

This work describes altered gene expression profiles in ventral midbrain dopamine and GABA neurons with aging. Experiments used two novel cell-type–specific reporter models to enable translatome analysis. Common age-driven alterations included increased inflammatory and prosurvival cell signaling and downregulation of synaptic transmission and plasticity genes. In individual cell types, we observed upregulation of serotonergic synthesis in GABA neurons and downregulation of mitochondrial function genes in dopamine neurons. Sex differences were detected in both neuronal types but were more prominent in dopamine neurons. These results reinforce aging as a risk factor for neurodegeneration in these neuronal populations while providing insight into potential mechanisms of homeostatic regulation during healthy aging and into genetic adaptations that are sex or neuron-type specific.

## Introduction

Biological aging is fundamentally variable. Humans and laboratory rodents exhibit great variance in cognitive abilities as they age (Spilich and Voss, 1983; Burger et al., 2007; Matzel et al., 2008), and individual tissues and cell types exhibit a wide range of responses to aging and its hallmarks (Hou et al., 2019; Allen et al., 2023; Jin and Cai, 2023; Kilfeather et al., 2024; Yaghmaeian Salmani et al., 2024). Brain aging is a leading risk factor for neurodegeneration (Anderton, 1997; Wyss-Coray, 2016; Hou et al., 2019) and likely plays a causative role in the development of diseases such as Alzheimer's (AD) (Wrigglesworth et al., 2021) and Parkinson's disease (PD; Nussbaum and Ellis, 2003; Poewe et al., 2017). Although findings in transcriptomic changes across the lifespan reveal selective sensitivity of regional cell populations to specific neurodegenerative diseases (Gonzalez-Velasco et al., 2020; Allen et al., 2023; Hahn et al., 2023), detailed mechanisms behind aging-related susceptibility or resilience to neurodegeneration are not well understood, especially within vulnerable brain regions.

Neurons of the ventral midbrain that synthesize and release dopamine may be particularly susceptible to aging and have been described as “biomarkers of aging” whose functional decrements may reflect a “core mechanism of aging itself” (Rollo, 2009). While multiple subtypes have been described, dopamine cell bodies can be roughly divided between the substantia nigra pars compacta (SNc), which projects through the nigrostriatal pathway and is necessary for the initiation of voluntary movement, and the ventral tegmental area (VTA), which projects widely through the mesocorticolimbic pathway to regulate cognitive, motivational, and affective behaviors (Schultz, 2002; Chaudhury et al., 2013). Nigrostriatal function naturally decreases with age (Branch et al., 2014; Howell et al., 2020; Noda et al., 2020), and motor impairment due to dopamine neurodegeneration is a hallmark of PD (Jankovic, 2008; Trist et al., 2019; Ledonne et al., 2023). Conversely, recent work in mice has selectively implicated VTA dopamine neurons in the development of AD (Nobili et al., 2017; Blankenship et al., 2024; Spoleti et al., 2024).

While subsets of dopamine neurons also noncanonically release the inhibitory neurotransmitter GABA (Tritsch et al., 2012), a nondopaminergic midbrain population of vesicular GABA transporter (VGAT)-expressing neurons serves as both local interneurons and projection cells (Reiner et al., 1998; van Zessen et al., 2012; Ntamati and Lüscher, 2016; Yoo et al., 2016; Nagaeva et al., 2020). Although GABA neurons demonstrate relative resiliency (Rissman et al., 2007), recent longitudinal studies point to a global decrease of GABA with aging in multiple regions of the brain (Zuppichini et al., 2024). GABA has broad effects due to its role as the brain's principle fast inhibitory neurotransmitter, and decreased GABA signaling has been associated with the development of neurodegenerative diseases (Błaszczyk, 2016; Purves-Tyson et al., 2021; Bang et al., 2023). Particularly in AD, alterations of the balance between excitatory and inhibitory signaling could contribute substantially to cognitive decline (Rissman and Mobley, 2011). Conversely, modulatory increase of GABA availability can ameliorate the effects of age in midbrain auditory neurons (Brecht et al., 2017). Hence, understanding the effects of aging on both GABA and dopamine neurons is essential due to their complex interactions in the ventral midbrain and the critical role of dysfunctional dopamine release in age-related neurodegenerative diseases.

**Figure 1.**
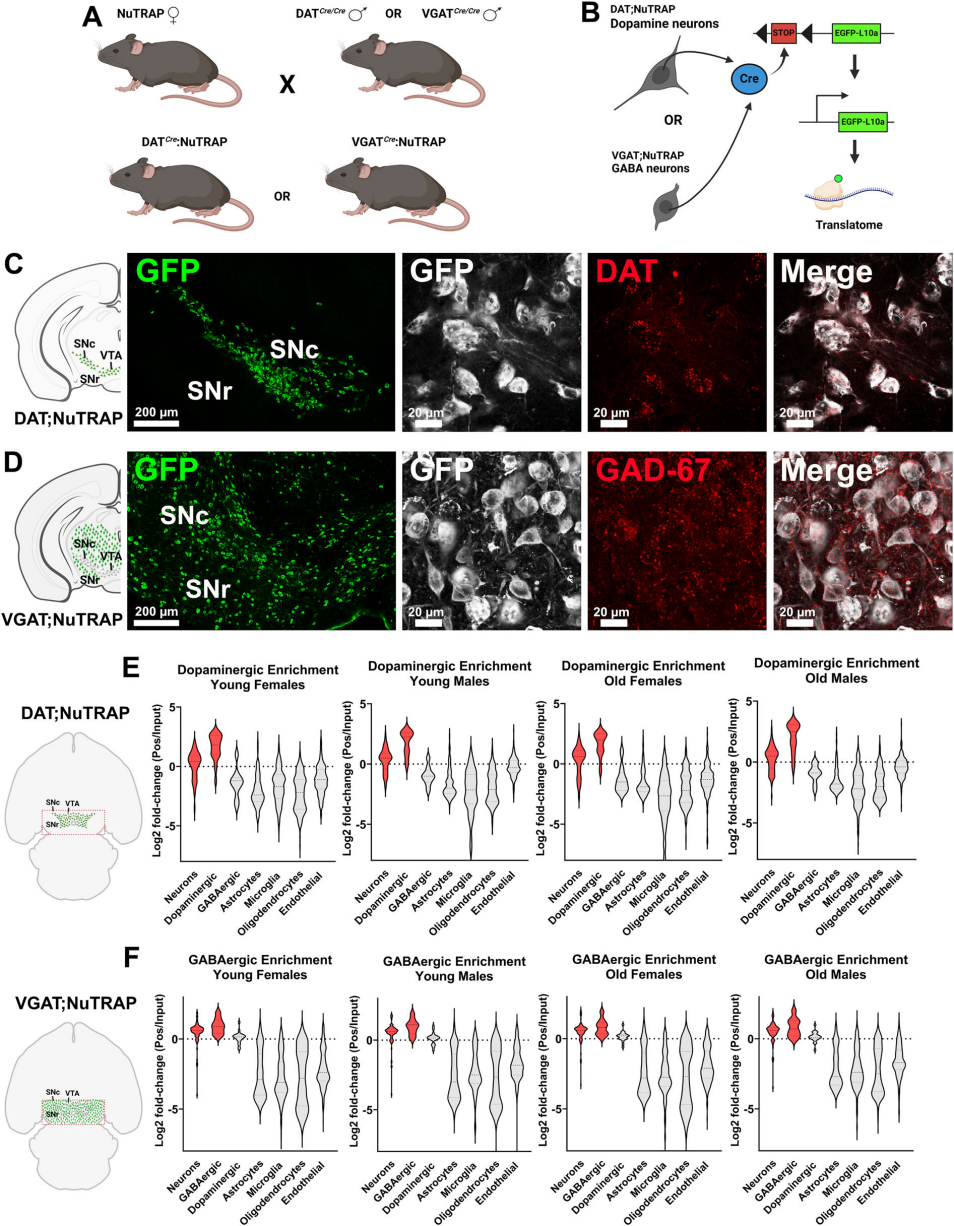
Cell-type–specific NuTRAP mouse models. ***A***, DAT;NuTRAP and VGAT;NuTRAP mice were generated by crossing NuTRAP mice with DAT-Cre or VGAT-Cre mice. ***B***, In the presence of *Cre-recombinase*, the floxed stop codon was removed, and ribosomal subunit L10a fused with GFP was expressed in a cell-type–specific manner. ***C***, A representative image of DAT;NuTRAP mice; GFP signal was detected in dopamine transporter (DAT) expressing cells. ***D***, A representative image of VGAT;NuTRAP mice; GFP signal was detected in GAD-67 expressing cells. No qualitative difference was observed between the GFP signal of young or old mice. SNc, substantia nigra pars compacta; VTA, ventral tegmental area; SNr, substantia nigra pars reticulata. ***E***, The region of the midbrain collected during GFP-tagged dopaminergic mRNA isolation (red-dotted rectangle) and gene enrichment results of the RNA-seq dataset for DAT;NuTRAP mice, separated by age and sex. ***F***, The region of the midbrain collected during GFP-tagged GABAergic mRNA isolation (red-dotted rectangle) and gene enrichment of the RNA-seq dataset for VGAT;NuTRAP mice, separated by age and sex. Neuronal and dopaminergic (DAT;NuTRAP) or GABAergic (VGAT;NuTRAP) enriched genes are represented in red.

To elucidate the impacts of healthy aging on the translatome of midbrain dopaminergic and GABAergic neurons, here we combined two transgenic tools by crossing neuron-type–specific *Cre-recombinase*–expressing mice (either DAT-Cre or VGAT-Cre) with mice expressing a *Cre*-dependent “translating ribosome affinity purification” (TRAP) transgene (Fig. 1*A*). Expression of the TRAP transgene resulted in expression of GFP-tagged ribosomal subunit L10a, in a *Cre-*dependent manner (Fig. 1*B*). In the crosses used in the present study, the TRAP transgene was expressed only in dopamine neurons (DAT-Cre) or GABA neurons (VGAT-Cre) allowing for cell-type–specific isolation of ribosome-bound, actively translating messenger RNAs (mRNA). TRAP maintains an advantage over bulk RNA sequencing in that it targets actively translating RNA from a cell-type–specific population instead of total RNA, thus more closely matching protein expression (Roh et al., 2017; Blevins et al., 2019; Chucair-Elliott et al., 2020; Ocanas et al., 2022; Kilfeather et al., 2024). Additionally, TRAP affords cell-type specificity without the confounds of cell sorting (Tiklova et al., 2019; Ocanas et al., 2022). By comparing midbrain dopamine and GABA neurons, we identified that these cells undergo some similar changes in regulation, particularly an increase of inflammatory responses and upregulation of prosurvival pathways. We also found that both cell types show downregulation of genes involved in synaptic connectivity and plasticity. Cell-type–specific effects included a surprising upregulation of serotonergic synthesis in GABA neurons, as well as a significant downregulation of genes connected to mitochondrial function and calcium signaling in dopaminergic neurons. Furthermore, we detected that changes in translatomic profiles with aging were different between males and females, a feature that was more prominent in dopamine neurons. Overall, the data provide a detailed description of age-driven molecular effects in both dopamine and GABA neurons from a single brain area (the ventral midbrain) and could be useful in targeting the biological consequences of aging in these cell populations.

## Materials and Methods

### Animals

Adult male and female DAT^IRES*cre*^ (RRID:IMSR_JAX:006660; Bäckman et al., 2006), Vgat-ires-cre knock-in (C57BL/6J; RRID:IMSR_JAX:028862; Vong et al., 2011), and NuTRAP (RRID:IMSR_JAX:029899; Roh et al., 2017) mice were originally obtained from The Jackson Laboratory. These mice were group housed and mated at the Oklahoma Medical Research Foundation (OMRF) Comparative Medicine facilities, on a 12 h light/dark cycle. To generate “DAT;NuTRAP” mice, DAT^IRES*cre*^ (cre/cre) males were mated with homozygous NuTRAP^Flox/Flox^ females, and “VGAT;NuTRAP” mice were generated by mating Vgat-ires-cre (cre/cre) males with homozygous NuTRAP^Flox/Flox^ females (Fig. 1*A*,*B*). The first two progenies of each breeding pairs were genotyped, in accordance with the Jackson suggested PCR protocol, for both respective-*Cre* and *Flox* genes (see Table 1 for specific primer sequences). In addition, sporadic genotyping throughout the fertile life of each pair was performed to ensure accurate genotypes. Experimental mice were aged naturally within our colony. All mice in the “Old” group were 21 months old, with the exception of one VGAT;NuTRAP female, which was 24 months old (Table 2). Previous aging work in mice has identified 18–25 months as a range of altered gene expression and declining brain function, including in dopamine neurons (Branch et al., 2014; Masser et al., 2017; Howell et al., 2020; Kellogg et al., 2023; Ocañas et al., 2023; Troyano-Rodriguez et al., 2023). The age of choice (21 months) was intended to target neuronal translatomic profile changes in mostly healthy mice prior to the development of age-related health issues. Mice that were beyond life expectancy (>28 months) were not studied because of the potential for survival bias, as populations with exceptional longevity (e.g., centenarians in humans) are thought to possess factors that diverge from normal aging (Willcox et al., 2006; Fischer et al., 2016; Trivedi et al., 2024). For the “Young” group, 17 out of 20 mice were aged 8–9 months old (Table 2), with a few exceptions required to complete the cohorts due to uneven litter sizes and sex distributions. All animal procedures were performed in accordance with the OMRF Institutional Animal Care and Use Committee.

**Table 1.** Primers used for used for mouse genotyping and antibodies used for immunofluorescence

Reagent	Source	Identifier
Oligo: TGGCTGTTGGTGTAAAGTGG	Sequence: JAX/reagent, Integrated DNA Technologies (IDT)	DATIREScre, forward
Oligo: GGACAGGGACATGGTTGACT	Sequence: JAX/reagent, IDT	DATIREScre, reverse, WR
Oligo: CCAAAAGACGGCAATATGGT	Sequence: JAX/reagent, IDT	DATIREScre, reverse, TG
Oligo: CTTCGTCATCGGCGGCATCTG	Sequence: JAX/reagent, IDT	Vgat-ires-cre, forward
Oligo: CAGGGCGATGTGGAATAGAAA	Sequence: JAX/reagent, IDT	Vgat-ires-cre, reverse, WR
Oligo: CCAAAAGACGGCAATATGGT	Sequence: JAX/reagent, IDT	Vgat-ires-cre, reverse, TG
Oligo: GACCACCTACAAGGCCAAGA	Sequence: JAX/reagent, IDT	NuTRAPFlox/Flox, forward, WT
Oligo: CCAGTTTGGCAATGTCTTCA	Sequence: JAX/reagent, IDT	NuTRAPFlox/Flox, reverse, WT
Oligo: CTGGCTTCTGAGGACCG	Sequence: JAX/reagent, IDT	NuTRAPFlox/Flox, forward, TG
Oligo: AGCCTGCCCAGA AGACTCC	Sequence: JAX/reagent, IDT	NuTRAPFlox/Flox, reverse, TG
Antibody: Goat anti-GFP (1:200)	Novus	Catalog #NB100-1770/RRID: AB_10128178
Antibody: Rat anti-DAT-Nt (1:1,000)	Millipore	Catalog #MAB369/RRID: AB_2190413
Antibody: Rabbit anti-GAD67 (1:100)	Thermo Fisher Scientific	Catalog #PA5-21397/RRID: AB_11153284
Antibody: Donkey anti-goat Alexa Fluor 488 conjugate (1:500)	Jackson ImmunoResearch Laboratories	Catalog #705-545-147/RRID: AB_2336933
Antibody: Donkey anti-rabbit Alexa Fluor 594 conjugate (1:500)	Jackson ImmunoResearch Laboratories	Catalog #711-585-152/RRID: AB_2340621
Antibody: Donkey anti-rat Alexa Fluor 647 conjugate (1:500)	Jackson ImmunoResearch Laboratories	Catalog #712-605-150/RRID: AB_2340693

**Table 2.** Specific ages of all animals used in the study, by group

Animal_ID	Age	Animal_ID	Age	Animal_ID	Age	Animal_ID	Age
DAT-Cre; NuTRAP							
Young_Male_1	10.5 months	Old_Male_1	21 months	Young_Female_1	9 months	Old_Female_1	21 months
Young_Male_2	8 months	Old_Male_2	21 months	Young_Female_2	9 months	Old_Female_2	21 months
Young_Male_3	9 months	Old_Male_3	21 months	Young_Female_3	9 months	Old_Female_3	21 months
Young_Male_4	9 months	Old_Male_4	21 months	Young_Female_4	9 months	Old_Female_4	21 months
Young_Male_5	9 months	Old_Male_5	21 months	Young_Female_5	8 months		
VGAT-Cre; NuTRAP							
Young_Male_1	9 months	Old_Male_1	21 months	Young_Female_1	9 months	Old_Female_1	24 months
Young_Male_2	8.5 months	Old_Male_2	21 months	Young_Female_2	9 months	Old_Female_2	21 months
Young_Male_3	7 months	Old_Male_3	21 months	Young_Female_3	9 months	Old_Female_3	21 months
Young_Male_4	7 months			Young_Female_4	8.5 months	Old_Female_4	21 months
				Young_Female_5	8.5 months	Old_Female_5	21 months
				Young_Female_6	8.5 months		

### TRAP transgene and generation of NuTRAP crosses

NuTRAP mice (Roh et al., 2017) express a transgene construct targeted to the Rosa26 locus that drives expression of the 60S ribosomal subunit L10a fused to EGFP (Fig. 1*B*). Upstream to the construct is a double floxed stop codon (loxP-stop-loxP), which is excised in the presence of *Cre-recombinase*. The expression of this transgene results in the formation of GFP-tagged polysomal complexes that can be isolated and purified with the use of anti-GFP antibodies (Abcam, catalog #ab290) and protein G magnetic beads (Invitrogen, catalog #10003D). When crossed with mice that express *Cre-recombinase* in a cell-type–specific manner, GFP-tagged ribosomal subunits are expressed only in cells expressing *Cre*. DAT^IRES*cre*^ are transgenic mice that express *Cre* under the promoter of the dopamine transporter gene (*Slc6a3*; DAT). When crossed with NuTRAP mice (Fig. 1*A*), DAT;NuTRAP progeny exhibit GFP-tagged ribosomes only in cells expressing DAT. In a similar manner but in a different cell type, Vgat-ires-cre express *Cre* under the promoter for the vesicular GABA transporter gene (*Slc32a1*; VGAT). VGAT;NuTRAP mice (Fig. 1*A*), therefore, have GFP-tagged ribosomes only in cells expressing VGAT. The ability to isolate GFP-tagged polysomal complexes in a *Cre*-dependent manner allows for valuable translatomic studies in individual cell types, even in complex tissue such as the brain (Chucair-Elliott et al., 2020).

### Histology processing and immunofluorescence

Cell-type specificity of the GFP-tagged ribosomes was assessed by immunofluorescence imaging for both young and old animals. Representative images from DAT;NuTRAP and VGAT;NuTRAP brains are shown in Figure 1, *C* and *D*, respectively. At killing, mice received an intraperitoneal injection of 2,2,2-tribromoethanol (Sigma-Aldrich, catalog #T48402-25G, 0.25 g/kg). Mice then underwent cardiac perfusion with 10% sucrose (Sigma-Aldrich, catalog #S7903-5KG), followed by 4% paraformaldehyde (PFA; Sigma-Aldrich, catalog #158127-500G) in 1× phosphate-buffered saline (PBS; Fisher Bioreagents, catalog #BP399-1), using a Perfusion Two automated pressure perfusion system (Leica Biosystems). After harvest, the entire brain was placed in 4% PFA for 24 h and then in 30% sucrose and stored in 4°C until embedding. Tissues were embedded in OCT compound (Sakura Finetek, catalog #4583), and 50 µm coronal slices were sectioned with a CryoStar NX70 Cryostat (Thermo Fisher Scientific). Brain slices were maintained in Cryoprotectant 3 (30% sucrose, 30% ethylene glycol, 1% PVP-40, in 0.1 M PBS) until time of staining. At staining, free-floating brain slices were washed with 1× PBS, for removal of the cryoprotectant, and then permeabilized with 0.2% Triton X-100 (Sigma-Aldrich, catalog #T8787) in 1× PBS (PBS-T) for 1 h (four times, 15 min). After PBS-T, slices were blocked for an hour with 7% normal donkey serum (NDS; Jackson ImmunoResearch Laboratories, catalog #017-000-121) prepared in 0.2% PBS-T. Table 1 lists all antibody information and dilutions. Incubation with primary antibodies and 7% NDS was performed at 4°C for 72 h, after which samples were washed with 0.2% PBS-T for 1 h (four times, 15 min). Incubation with secondary antibodies and 7% NDS was performed at room temperature (24°C) for 2 h, after which slices were washed in 0.2% PBS-T (two times, 15 min) and 1× PBS (two times, 15 min). Brains slices were then transferred to coated slides (Globe Scientific, catalog #1358P), mounted with ProLong Glass Antifade mounting media (Invitrogen, catalog #P36980) and covered with glass coverslips (Fisherbrand, catalog # 12-545). Immunofluorescence images were obtained at the Imaging Core Facility at OMRF, using a LSM 710 confocal microscope (Carl Zeiss). Individual channel images were consistently (for all groups) adjusted for brightness and contrast when necessary.

### Sample collection for RNA

Mice were anesthetized with isoflurane and rapidly decapitated. To minimize activity-induced transcriptomic changes, we immediately removed the brain and placed it in ice-cold choline chloride cutting solution, containing the following (in mM): 110 choline chloride; 2.5 KCl; 1.25 Na_2_PO_4_; 0.5 CaCl_2_; 10 MgSO_4_; 25 glucose; 11.6 Na-ascorbate; 3.1 Na-pyruvate; 26 NaHCO_3_; 12 *N*-acetyl-l-cysteine; and 2 kynurenic acid. Using a vibrating microtome (Leica VT1200s), 600-µm-thick horizontal brain slices were collected, accounting for the entire rostral–caudal length of the ventral midbrain. Sections were moved onto a prechilled collection block, where the surrounding tissue (such as the pons, hippocampus, and cortex) was removed. Remaining bilateral midbrain portions (containing the SNc and the VTA) were collected into Eppendorf tubes and flash-frozen for RNA preservation. All samples were stored in −80°C freezer until the time of TRAP and mRNA isolation (Heiman et al., 2014).

### TRAP and mRNA extraction

TRAP isolation was carried out as previously reported (Heiman et al., 2014; Chucair-Elliott et al., 2020; Ocanas et al., 2022). Briefly, midbrain slices were placed in 200 µl of ice-cold homogenization buffer (50 mM Tris; 12 mM MgCl_2_; 100 mM KCl; 1% NP-40; 1 mg/ml sodium heparin; 1 mM DTT), pH 7.4, supplemented with 100 μg/ml cycloheximide (Millipore, catalog #C4859-1ML), 200 units/ml RNaseOUT Recombinant Ribonuclease Inhibitor (Thermo Fisher Scientific, catalog #10777019), and 1× cOmplete, EDTA-free Protease Inhibitor Cocktail (Millipore, catalog #11836170001). Homogenization used a cordless motor pestle (Kimble #749520-0090 and #749540). After initial homogenization, an additional 500 µl of buffer was added to the samples, washing the pestle in between pulses. Homogenate solution volume was then brought to a total of 1.5 ml and centrifuged at 12,000 × *g* for 10 min at 4°C. After centrifugation, 100 µl of the supernatant was removed and set aside on ice (“input fraction”). The remaining supernatant (∼900 µl of “positive fraction”) was transferred into a fresh tube and incubated with 1 µl anti-GFP antibody (Abcam, catalog #ab290). Both input and positive samples were placed in an end-over-end rotating mixer and incubated for 1 h at 4°C. Prewashed Dynabeads protein G (Invitrogen, catalog #10003D; 30 µl per sample) were then added to the positive fraction, and the final solution was incubated overnight (∼16 h) in a rotating mixer at 4°C. Positive tubes were then placed in a DynaMag-2 magnet and supernatant removed and saved as the “negative fraction.” The magnetic beads bound with GFP-labeled polyribosomes/mRNAs were washed with high-salt buffer (50 mM Tris; 12 mM MgCl_2_; 300 mM KCl; 1% NP-40; 100 μg/ml cycloheximide; 2 mM DTT), pH 7.5, three times. After the final wash, Dynabeads were separated from the GFP-labeled polyribosomes/mRNAs by adding 350 µl of Buffer RLT (QIAGEN) and 3.5 µl of 2-β mercaptoethanol, proceeding with incubation in a benchtop ThermoMixer (Eppendorf) for 10 min at 22°C. The eluted solution (“positive fraction”), free of magnetic beads, was placed in a fresh tube. mRNA isolation was carried out using an RNeasy Mini kit (#74104, QIAGEN), following manufacturer's suggested protocol. Isolated mRNA was then quantified with a NanoDrop spectrophotometer (Thermo Fisher Scientific, model ND-ONEC-W) and its quality measured in a 4150 TapeStation analyzer (Agilent, model G2992AA) using HSRNA ScreenTape (Agilent, catalog #5067-5579).

### Library construction and RNA sequencing

Directional mRNA libraries were prepared using NEBNext Ultra II Kit for Illumina [New England Biolabs (NEB), catalog #E7760L] in accordance to the manufacturer's directions and following previously established protocol (Chucair-Elliott et al., 2020; Ocanas et al., 2022). In summary, each sample of both positive and input fractions (ranging 3.5–35 ng of mRNA) were individually captured by poly-A RNA using NEBNext Poly(A) mRNA Magnetic Isolation Module (NEB, catalog #NEBE7490L). mRNA was then eluted from oligo-dT beads, fragmented using a thermal cycler at 94°C for 15 min, and first and second strands of cDNA were individually synthesized following manufacturer's guidance (NEB, catalog #E7760L). The final double-stranded cDNA was purified with the use of SPRISelect.

Beads (Beckman Coulter, catalog #B23318) are eluted in 50 µl 0.1× TE buffer. Next, adaptor ligation was performed using 100-fold dilution of the NEBNext adaptor in dilution buffer (NEB, catalog #E6609L), and PCR of the ligated products was conducted using the NEBNext Ultra II Q5 Master Mix (NEB, catalog #7760L) and unique index primers (NEB, catalog #E6609L), both provided with aforementioned kits, in accordance to manufacture's protocol (14 cycles). Purified libraries were then quantified using HS dsDNA Qubit kit (Thermo Fisher Scientific, catalog #Q33230). Library integrity, verified in a 4150 TapeStation analyzer (Agilent, model G2992AA) using HS D1000 ScreenTape (Agilent, catalog #5067-5584), revealed an average peak size of 332 bp. Libraries for each sample were then pooled at 5 nM concentration and sequenced by the OMRF Clinical Genomics Center using the Illumina NovaSeq 6000 system (S4 PE150). Data from this publication have been deposited in NCBI's Gene Expression Omnibus (Edgar et al., 2002) and are accessible through GEO Series accession number GSE295369.

### RNA-seq analysis

Using a high-performance computing cluster, fastq files were submitted to prealignment quality control (QC) analysis (Ewels et al., 2016), after which adaptors were removed and reads were trimmed for poly-G and *Q* < 20 (Krueger et al., 2023). Reads were aligned to the mouse genome assembly mm39 using STAR v2.7.10b (Dobin et al., 2013; Dobin, 2022), and quantification was performed using featureCounts (Liao et al., 2014), and post alignment QC reports were generated. Low read counts (read counts, <10) were removed from the count matrix using RStudio v.4.3.2 (Gentleman et al., 2004; RStudio: Integrated Development Environment for R, 2022), prior to differential analysis. Normalization and differential analysis was performed with DESEq2 (Love et al., 2014; R/Bioconductor; Gentleman et al., 2004) and Benjamini–Hochberg multiple testing correction. All remaining data processing and plot generation were performed in RStudio v.4.3.2. Data processing and analysis were supported by the OMRF Center for Biomedical Data Sciences.

### Enrichment analysis

To confirm cell-type–specific enrichment of the positive fractions, DESEq2 analysis was used to compare gene expression between positive (condition) and input (control) fractions. Using RStudio, the resulting count matrices were filtered for a predefined list of genes (GEO repository accession number GSE295369) for detection of neuronal (Mckenzie et al., 2018), dopaminergic, GABAergic, astrocytic, microglial, oligodendrocytic, and endothelial specific genes (Mckenzie et al., 2018). Samples were separated by cell-type (dopaminergic and GABAergic), sex (males and females), and age (young and old), and each group analysis performed individually. Resulting Log2 fold-change between positive and input fractions (Fig. 1*E*,*F*) was plotted using GraphPad Prism v.10 [GraphPad Prism (Version 10), 2024].

### Differential expression analysis for aging

Postalignment raw count matrices were processed prior to differential expression analysis, using RStudio, in the following manner: (1) removal of genes with low counts and (2) removal of astrocytic and microglial specific genes (Mckenzie et al., 2018; GSE295369) batch correction for (a) collection date, (b) sequencing date, (c) duplication levels, and (d) millions of reads. DESeq2 normalization and differential analysis allowed for comparison of old animals (condition) versus young animals (control). Overall aging analyses for DAT;NuTRAP (*n* = 10 young and 9 old) and VGAT;NuTRAP (*n* = 10 young and 8 old) were performed independently. Aging analysis was also evaluated in a sex-separate manner between old females (condition) and young females (control; DAT;NuTRAP *n* = 5 young and 4 old; VGAT;NuTRAP *n* = 6 young and 5 old) and between old males (condition) and young males (control; DAT;NuTRAP *n* = 5 young and 5 old; VGAT;NuTRAP *n* = 4 young and 3 old).

### Gene ontology and ingenuity pathway analysis

Using RStudio, the differentially expressed genes (DEGs) for each comparison (cutoff Log2FC = |0.5|; *p*_Adj_ < 0.5) were processed and analyzed for changes in biological processes using Kyoto Encyclopedia of Genes and Genomes (KEGG; Kanehisa, 2000) and Gene Ontology (GO; Drabkin et al., 2015; Ashburner et al., 2000; Aleksander et al., 2023) pathway analysis. Statistical significance for KEGG and GO were *p* < 0.05 and *q* < 0.2. DAT;NuTRAP mice were further analyzed for synaptic ontology pathways using the SynGO platform (Koopmans et al., 2019). DESeq2 results were also uploaded into the Qiagen Ingenuity Pathway Analysis (Ingenuity Pathway Analysis (IPA), 2024) for further understanding of upregulated and downregulated pathways and for comparison between gene regulation in different groups (females vs males and DAT;NuTRAP vs VGAT;NuTRAP).

## Results

### NuTRAP crosses successfully provide translatomes enriched for midbrain cell types

To verify that both “Old” and “Young” NuTRAP crosses (DAT;NuTRAP and VGAT;NuTRAP) express GFP ribo-tags in a cell-type–specific manner, we stained PFA-fixed midbrain slices with antibodies targeting GFP and DAT, in DAT;NuTRAP mice or GAD-67 (glutamate decarboxylase, the enzyme essential for synthesis of GABA; Soghomonian and Martin, 1998) in VGAT;NuTRAP mice. In DAT;NuTRAP mice, we observed GFP staining in areas corresponding to the SNc and VTA (Fig. 1*C*), and as expected the GFP signal overlapped DAT puncta in dopaminergic neurons. We observed a broader staining of GFP in VGAT mice, as expected given the multiple types of GABA neurons present in the ventral midbrain (Fig. 1*D*). The GFP signal in VGAT;NuTRAP mice also overlapped with the GAD-67 staining present in the cell bodies and processes of GABA neurons. Hence, immunofluorescence experiments confirmed that both young and old mice express GFP-tagged ribosomes in a neuron-type–specific manner.

After confirming that all mice express GFP-tagged ribosomes, we purified the GFP-bound ribosomal complexes and extracted the mRNA present in these complexes (“positive fraction”). For comparison purposes, we also extracted RNA from bulk midbrain homogenate set aside prior to GFP purification (“input fraction”). We synthesized and sequenced RNA libraries using both positive (GFP-bound mRNA) and input (bulk RNA) fractions. To confirm that the data obtained with the positive fractions reflected the expected cellular specificity, we then performed differential analysis comparing the RNA-seq results for the enriched GFP-positive and the input data (bulk RNA expression). Enrichment analysis for all DAT;NuTRAP groups (young females or males and old females or males) confirmed that the translatomes isolated from these mice were successfully enriched for dopaminergic neuronal transcripts (Fig. 1*E*; GSE295369). In accordance, differential analysis performed in VGAT;NuTRAP groups also demonstrated that samples from all four groups exhibit an enrichment of neuronal and GABAergic genes (Fig. 1*F*; GSE295369). Enrichments showed a consistent pattern across ages and sexes. The immunostaining and enrichment analyses are consistent with what has been described in the literature during model validation using NuTRAP mice or dopamine neuron-specific translatome analyses (Roh et al., 2017; Chucair-Elliott et al., 2020; Kilfeather et al., 2024). Thus we were confident the translatomics datasets are enriched for dopaminergic neurons (DAT;NuTRAP) or GABAergic neurons (VGAT;NuTRAP) and proceeded with differential analysis of aging and sex-biased gene expression.

### Orthogonal correlation with public datasets

**Figure 2.**
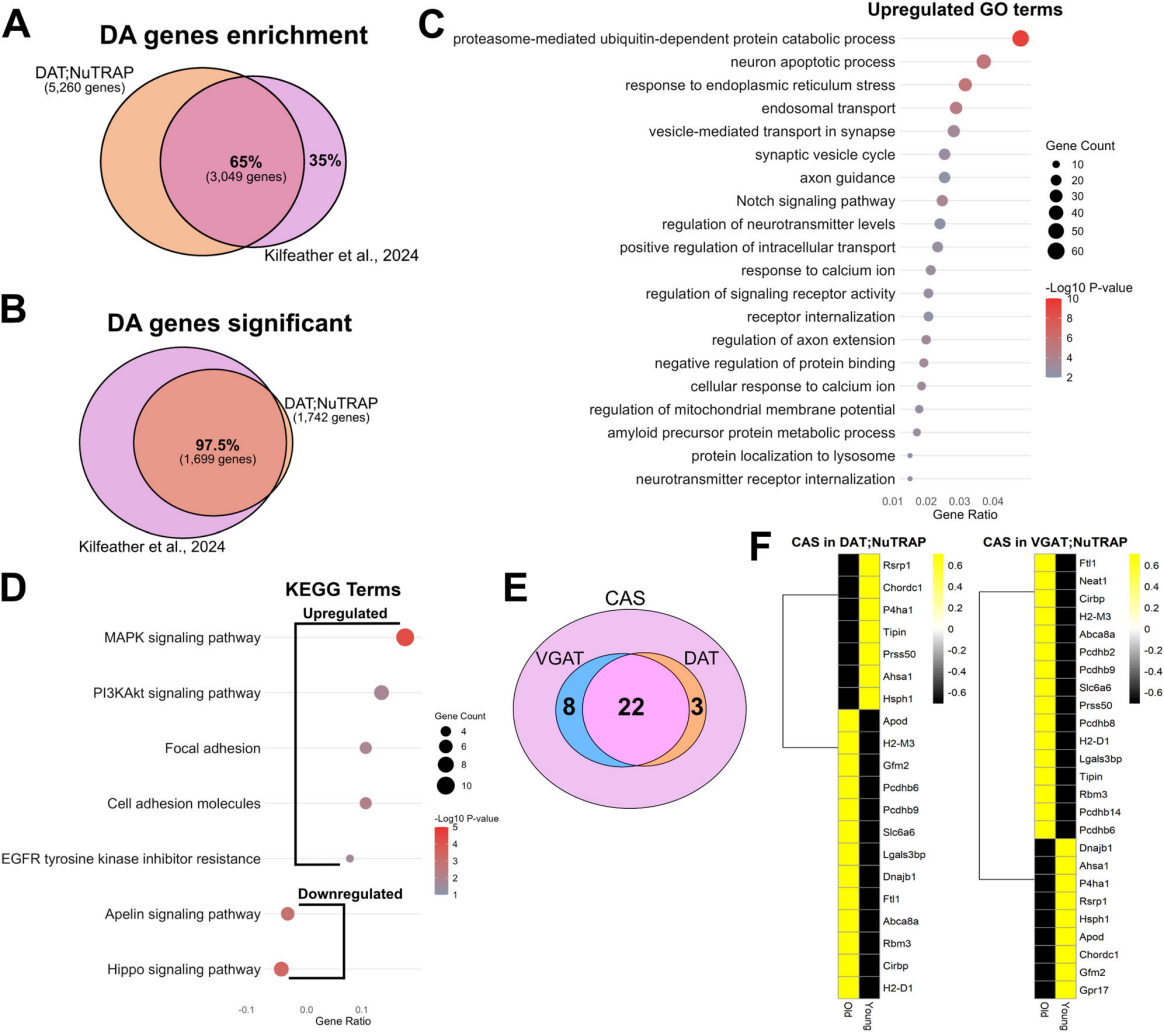
Orthogonal validation. ***A***, Comparison of dopaminergic gene enrichment between Kilfeather et al. (2024) and our dopamine dataset (all ages and sexes combined). ***B***, Overlap of significantly enriched genes between our dopaminergic dataset and Kilfeather et al. (2024). ***C***, GO terms of commonly upregulated genes with age [between our dataset and Kilfeather et al. (2024)]. ***D***, KEGG terms of commonly up- and downregulated genes with age [between our dataset and Kilfeather et al. (2024)]. Pathways analysis cutoff: *p* < 0.05 and *q* < 0.02. ***E***, The number of “CAS” (Hahn et al., 2023) genes present in the DAT;NuTRAP and VGAT;NuTRAP datasets and the number of genes common to both groups. ***F***, CAS genes up- (yellow) and down- (black) regulated in DAT;NuTRAP and VGAT;NuTRAP. Cutoff: Log2FC > |0.5|; adjusted *p* < 0.5.

In order to validate our findings on DAT;NuTRAP and VGAT;NuTRAP neuronal aging, we compared our translatomics data with previously published datasets obtained both with the TRAP technique (Kilfeather et al., 2024) and other transcriptomic techniques (Hahn et al., 2023). To validate the DAT;NuTRAP dopaminergic enrichment, we used published data from Kilfeather and colleagues (https://spatialbrain.org; Kilfeather et al., 2024), which used the correlation between TRAP RNA-seq and spatial transcriptomics to characterize the translatome profile of dopaminergic neurons in the midbrains of young and old mice. Spatial transcriptomics allowed for a detailed detection of gene expression changes in subpopulations of dopaminergic neurons and showed a high correlation with TRAP data. However, the TRAP approach demonstrated a greater detection power, offering the most inclusive and sensitive measure of dopaminergic gene expression. We started by analyzing our dopaminergic enrichment results (DESeq2: GFP-positive fraction × bulk input RNA-seq) with the same cutoff values (lfcThreshold = log_2_|1.05|; *p*_Adj_ < 0.01; Kilfeather et al., 2024). We found an overlap of 65% among the genes with expression above the threshold [lfc > log_2_(1.05); Fig. 2*A*; GEO repository accession number GSE295369], accounting for 3,049 genes in our DAT;NuTRAP positive fraction. We determined that the majority of the missing genes (35% of Kilfeather et al. enrichment) were removed from our dataset during low-count removal (read counts, <10) and therefore reflect the exclusion criteria used in this study. Comparing just the enriched transcripts with adjusted *p* < 0.01 (Pos > Input; GSE295369), we found an agreement of 97.5% of the genes showing significant enrichment [lfc > log_2_(1.05); *p*_Adj_ < 0.01] in both datasets (Fig. 2*B*). After confirming that the majority of the dopaminergic genes in our dataset agreed with those reported by Kilfeather et al., we compared the age-related changes represented in both datasets to perform independent GO and KEGG pathways analysis. Overlapping GO and KEGG terms (Fig. 2*C*,*D*; GSE295369) revealed agreement between changes detected in DAT;NuTRAP and some of the terms reported for protein–protein interaction of aging-related DEGs (Kilfeather et al., 2024), among them axonal extension, synaptic vesicle (endocytosis), and protein ubiquitination. Finally, we detected that all of the mitochondrial-related genes reported as downregulated (Kilfeather et al., 2024) are also downregulated in our dataset (GSE295369). Taken together, this analysis increased confidence in our dopaminergic aging dataset as it correlated closely with results from prior literature.

Using a combination of region-specific bulk RNA sequencing, spatial and single-nucleus transcriptomics, Hahn et al. (2023) created a detailed atlas of the mouse brain during aging analyzing several different brain sections. While aging-driven changes in gene expression are specific for each area of the brain, these authors detected the existence of a shared aging signature, termed “Common Aging Score, CAS,” which was present in all regions to varying degrees. Although a study of the midbrain was not included in this work, we hypothesized that a similar effect might occur in the area. We first removed microglial and astrocytic-specific genes (GSE295369, 42 in total; Mckenzie et al., 2018) from the CAS gene set (GEO repository accession number GSE295369). Next, we intersected the CAS with genes that are expected to be expressed in young dopamine and GABA neurons of the midbrain according to the Allen Brain Cell Atlas (https://portal.brain-map.org; Yao et al., 2023; Fig. 2*E*; GSE295369). We found 25 CAS genes expressed in dopamine neurons and 30 in GABA neurons, with 22 of the CAS genes expressed in both cell types. From this resultant CAS gene list (40 genes; GSE295369), we detected changes in the expression (old × young) of 20 out of 25 genes in our DAT;NuTRAP dataset and 25 out of 30 genes in the VGAT;NuTRAP (Fig. 2*F*; GSE295369). There was upregulation of 13 CAS genes in DAT;NuTRAP and 16 in VGAT;NuTRAP, accounting for a large majority of the CAS expected in both cell types. Among downregulated CAS genes, five genes were predicted to be present in dopamine and GABA cells (GSE295369; Allen Brain Cell Atlas: https://portal.brain-map.org; Yao et al., 2023) and were commonly downregulated in both DAT- and VGAT-expressing cells (Fig. 2*F*; *Asha1*, *P4ha1*, *Rsrp1*, *Hsph1*, and *Chordc1*); thus, they are likely to represent a common effect of aging in these cell types. Overall, our analysis detected that dopamine and GABA neurons of the midbrain are subjected to some of the signature alterations in CAS genes, in accordance with observations in other brain nuclei (Hahn et al., 2023).

### Differences and similarities of the aging process in midbrain dopamine and GABA neurons

**Figure 3.**
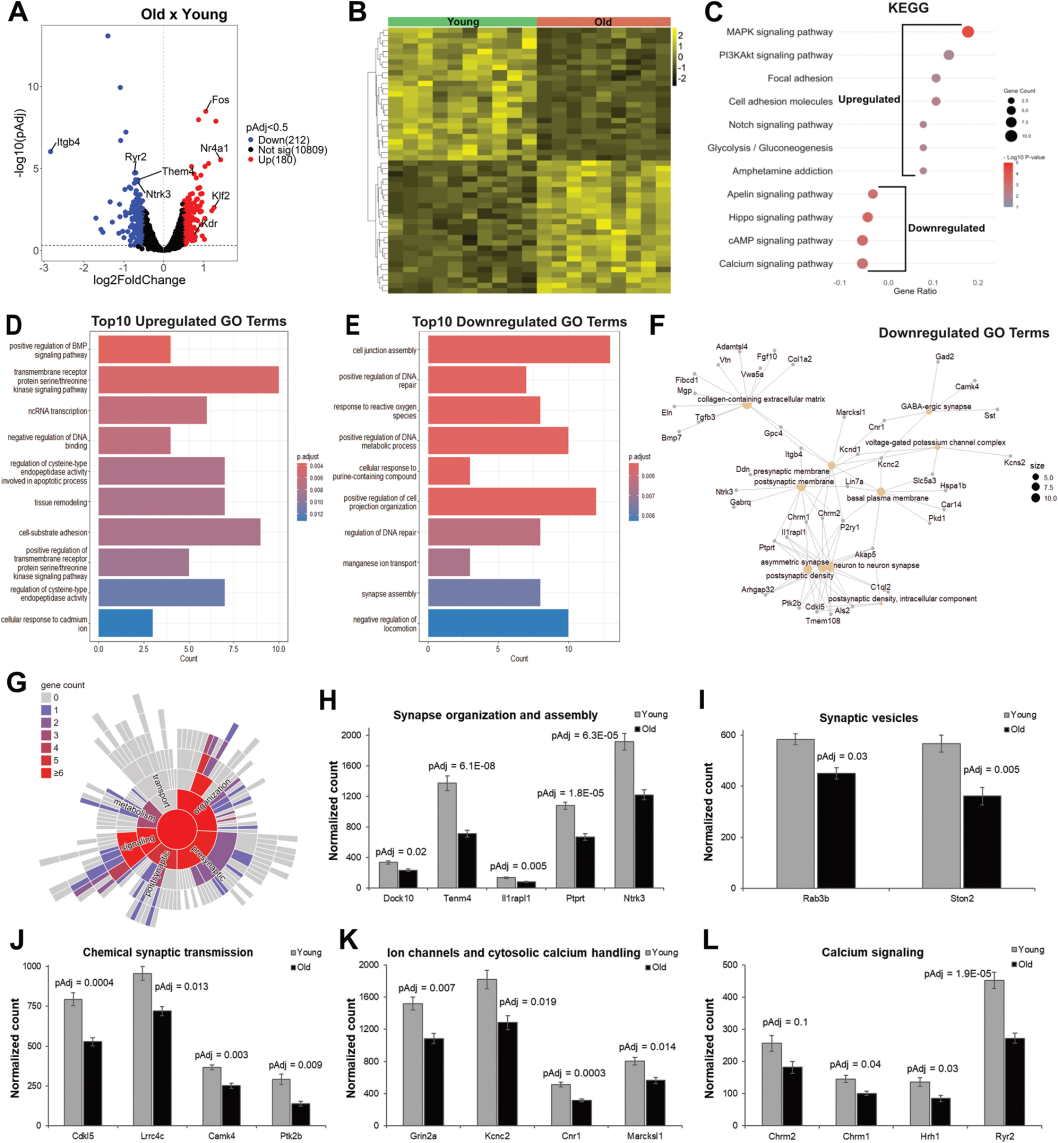
Gene expression changes with age in DAT;NuTRAP mice. ***A***, Volcano plot and (***B***) heatmap of differential expression analysis for DAT;NuTRAP mice. ***C***, KEGG pathway analysis showing most enriched terms in DAT;NuTRAP mice. Top GO terms of upregulated (***D***) and downregulated (***E***) genes in DAT;NuTRAP mice. ***F***, Gene-network analysis of downregulated genes in DAT;NuTRAP mice, showing several synaptic-related terms. ***G***, SynGO (Synapse Gene Ontology) analysis of synaptic-related DEGs in DAT;NuTRAP mice. ***H–K***, Synaptic-related genes and (***L***) calcium signaling-related genes significantly downregulated with age (adjusted *p* < 0.5).

Defining DEGs with aging as an adjusted *p* < 0.5 and log2FoldChange > |0.5|, we detected that age promoted upregulation of 180 genes and downregulation of 212 genes in dopamine neurons (Fig. 3*A*; GEO accession number GSE295369) and were consistently changed across all aged samples (Fig. 3*B*). KEGG analysis revealed that signaling pathways connected to inflammatory responses such as MAPK, Notch, and PI3K/Akt were all among the most upregulated terms with aging (Fig. 3*C*). Activation of MAPK signaling induces degeneration of dopaminergic neurons in the SNc (Mamais et al., 2022) and is upregulated in AD and PD patients (Ahmed et al., 2020). Notch signaling is activated in AD mouse models (Chen et al., 2019), and mutations involving Notch protein have been identified in AD patients (Kapoor and Nation, 2021). The PI3K/Akt signaling pathway is involved in different neuronal processes. Particularly under induced stress, PI3K/Akt exerts a cell-protective effect, enhancing expression of inflammatory cytokines and prosurvival signaling cascades (Hu et al., 2018; Razani et al., 2021). The activation of these pathways may suggest a neuronal response to increases in local, age-related inflammation, a phenomenon well studied in the cortex and hippocampus (Sparkman and Johnson, 2008; Starkey et al., 2012; Jurcau et al., 2024). Furthermore, KEGG analysis in combination with GO pathway analysis (Fig. 3*D*) showed upregulation of genes involved in cell adhesion and transmembrane signaling, indicating enhanced responses to the extracellular environment. Finally, the dataset points toward upregulation in genes connected with glycolysis and gluconeogenesis (Fig. 3*C*), which, in combination with the aforementioned decrease in mitochondrial-related genes, suggests an alteration in dopaminergic neuron metabolic regulation (Bender et al., 2006; Dai et al., 2023). Aging-related mitochondrial dysfunction in the brain is a widely studied topic (Bender et al., 2006; Stauch et al., 2014; Grimm and Eckert, 2017; Filograna et al., 2021) and is one of the main factors connecting aging of dopaminergic neurons and the occurrence of PD (Bose and Beal, 2016; González-Rodríguez et al., 2021; Moradi Vastegani et al., 2023).

KEGG analysis also revealed an expressive downregulation of genes involved in calcium and cAMP signaling, as well as hippo and apelin pathways (Fig. 3*C*). Apelin plays an important role in various neurological disorders (Cheng et al., 2012). Particularly in the SNc, apelin was shown to provide a protective effect against PD in rodent models (Haghparast et al., 2018; Zhu et al., 2020), and potential signaling downregulation suggests an increased vulnerability of the dopaminergic neurons with aging. KEGG terms in combination with GO pathways analysis (Fig. 3*E*) pointed to downregulation of DNA repair pathways as well as response to reactive oxygen species, providing additional insights into age-induced mechanisms of susceptibility in these neurons. A further look into the GO terms via network plot revealed downregulation of several synapse-related genes that participate in synaptic membranes, synaptic density, and transmission (Fig. 3*F*). Querying a list of 23 downregulated genes using the Syngo portal (syngoportal.org; Koopmans et al., 2019; Fig. 3*G*), we detected that the majority of synapse-related genes pointed to downregulation of terms associated with synapse organization and assembly (Fig. 3*H*) as well as crucial genes involved in dopaminergic transmission such as *Rab3b* (Chung et al., 2009; Fig. 3*I*) and *Cdkl5* (Jhang et al., 2020; Fig. 3*J*). Furthermore, several of the downregulated synaptic genes were involved in calcium regulation (Fig. 3*H*–*K*). In fact, we detected seven overlapping genes between downregulated synaptic genes and the ones identified by KEGG analysis as involved in calcium signaling (Fig. 3*C*): *Ntrk3*, *Camk4*, *Lrrc4c*, *Ptk2b*, *Grin2a*, *Kcnc2*, *Cnr1*, and *Marcksl1*. In addition, we also detected downregulation of important intracellular calcium regulators such as *Chrm1* (Fiorillo and Williams, 2000) and *Ryr2* (Bertan et al., 2020), pointing toward a change in intracellular calcium dynamics, a neuronal age effect widely described in the literature (Chan et al., 2007; Mattson, 2007; Toescu and Verkhratsky, 2007; Surmeier et al., 2010; Branch et al., 2014). Curiously, inhibition of ryanodine receptors (Ryr) provides a protective effect in situations of intracellular calcium dyshomeostasis (Huang et al., 2017) and could be identified as a potential self-protective mechanism against increases in free intracellular calcium, although its loss also affects synaptic plasticity by impairing remodeling of dendritic spines and decreasing excitatory synapses (Bertan et al., 2020). Overall, our analysis of age-related translatomic changes in dopaminergic neurons provided several insights into mechanisms of susceptibility and vulnerability of dopaminergic neurons such as the upregulation of inflammatory signaling and downregulation of mitochondrial genes and genes involved in synaptic transmission and plasticity. However, it also identified upregulation of prosurvival signaling, as well as changes in metabolic regulation and intracellular calcium dynamics, which are consistent with maintenance of homeostasis in the face of age-related deficits.

**Figure 4.**
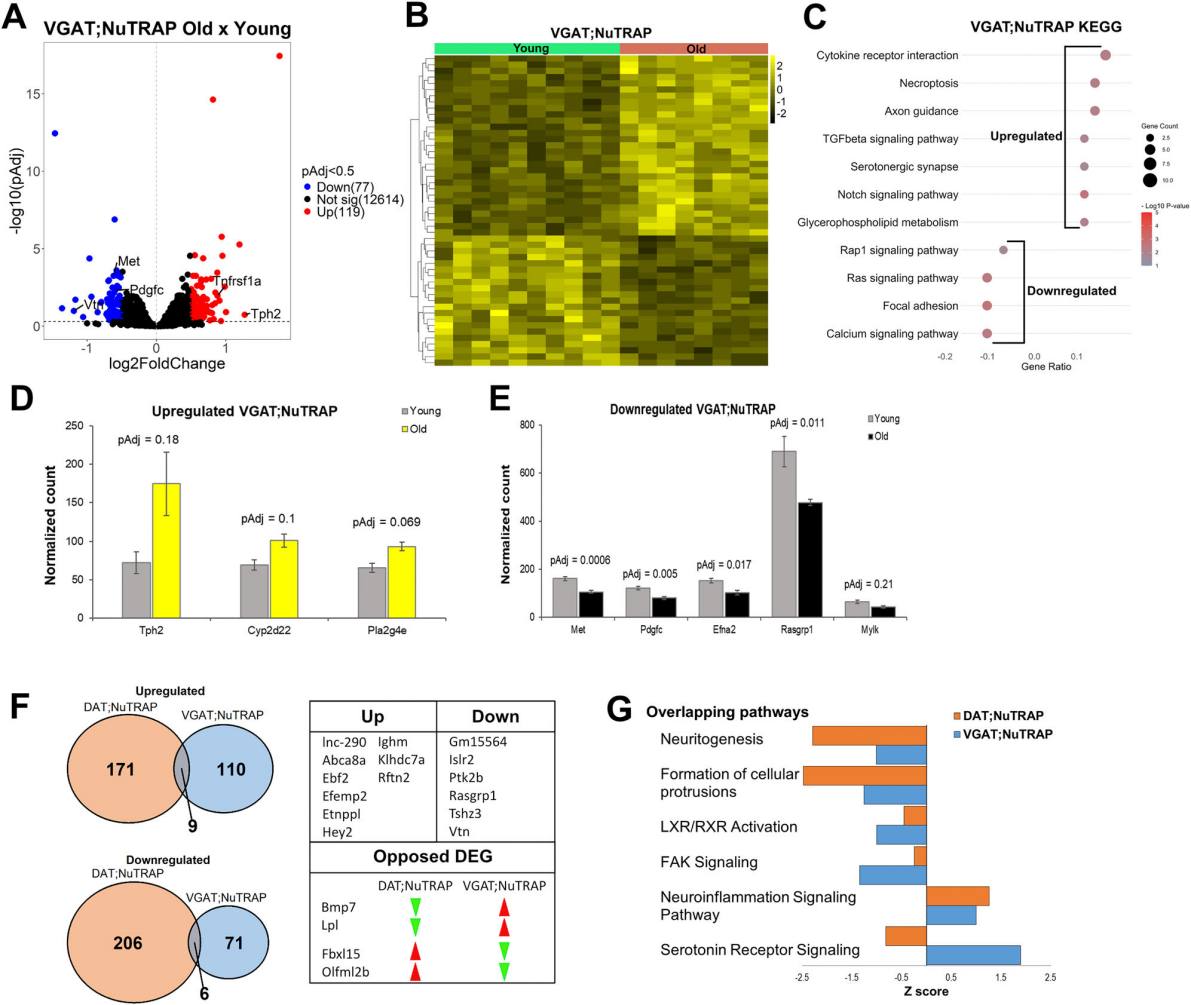
Gene expression changes with age in VGAT;NuTRAP mice and comparison between DAT and VGAT-expressing neurons. ***A***, A volcano plot and (***B***) heatmap of differential expression analysis for VGAT;NuTRAP mice. ***C***, KEGG pathway analysis showing most enriched terms in VGAT;NuTRAP mice. Serotonergic-related genes significantly upregulated (***D***; adjusted *p* < 0.5) and cell signaling-related significantly downregulated genes (***E***) in VGAT;NuTRAP mice. ***F***, Intersected up- and downregulated genes with age between both DAT;NuTRAP and VGAT;NuTRAP datasets. ***G***, IPA of overlapping pathways for both groups. Pathways analysis cutoff: *p* < 0.05 and *q* < 0.02.

To determine if GABA neurons share similar responses to aging as dopaminergic neurons, we also probed their translatomic profile. Analyzing age-driven changes in VGAT;NuTRAP mice, we detected upregulation of 119 and downregulation of 77 genes (Fig. 4*A*,*B*; GSE295369). Similar to results from DAT;NuTRAP mice, KEGG analysis returned terms associated with the upregulation of pathways involved in inflammation response (Fig. 4*C*) such as cytokine receptor interaction, TGFbeta, Notch signaling, and necroptosis. Some of the upregulated genes (Fig. 4*D*; GSE295369) were involved in serotonergic synthesis, including the serotonergic marker *Tph2* (Chen and Miller, 2012), *Pla2g4e*, and *Cyp2d22*, which is upregulated with age in other areas of the brain (Haduch et al., 2022). The increased expression of genes involved in the synthesis of serotonin in midbrain GABA neurons, which are not predicted to synthesize or release serotonin, was surprising and could reflect development of cotransmission throughout the lifespan. We subsequently confirmed the presence of these genes in midbrain GABA neurons using the Allen Brain Cell Atlas (https://portal.brain-map.org; Yao et al., 2023). *Tph2* and *Cyp2d22* were present at low levels in the midbrain, while *Pla2g4e* was highly expressed in several GABA-releasing cells (data not shown). Previously, glutamate corelease has been suggested to be neuroprotective in midbrain dopamine neurons and subject to age-related regulation (Buck et al., 2021b). Increased serotonin synthesis in GABA neurons may provide a second instance of midbrain neurons adapting their neurotransmitter repertoire with age and could convey homeostasis or neuroprotection through an undetermined mechanism. Furthermore, KEGG analysis pointed toward downregulation of genes involved in Rap1 and Ras signaling, calcium signaling, and focal adhesion (Fig. 4*C*,*E*). Similar to what we observed in DAT;NuTRAP mice, these terms are associated with synaptic transmission and plasticity (Ye and Carew, 2010; Stornetta and Zhu, 2011). In particular, we found that *Rasgrp1* (Fig. 4*E*), which is downregulated with age in the human cortex (Gonzalez-Velasco et al., 2020), is also downregulated with age in dopamine neurons (Fig. 2*G*) and is downstream from calcium signaling (Ebinu et al., 1998; Ye and Carew, 2010), which was found downregulated in both cell types (Figs. 3*C*, 4*C*).

Comparatively, dopamine and GABA neurons exhibited divergent sets of individual DEGs with aging (Fig. 4*F*), suggesting that these neurons establish unique age-related molecular phenotypes (Figs. 3*A*, 4*A*; GSE295369), as evidenced by the small number of shared up- and downregulated genes (Fig. 4*F*). One noteworthy difference between GABA and dopamine neurons is the predicted activation of serotonin receptor signaling pathway in GABA neurons, in opposition to the predicted decrease in dopamine neurons (Fig. 4*G*; GEO accession number GSE295369). However, the *Z*-scores for activated and inhibited overlapping pathways (Fig. 4*G*; GSE295369) suggest that the majority of the predicted biological changes in both neuron types remain the same: activation of neuroinflammation signaling pathways and decrease in pathways that correlate with synaptic function. Taken together, we conclude that aging promotes alterations in gene expression in midbrain dopamine and GABA neurons that are very different at first look (when considering DEGs) but that converge to parallel predicted biological pathways. Overall, common translatomic changes of aged midbrain dopamine and GABA neurons reflect an increased response to inflammatory and extracellular signaling, accompanied by a decrease in synaptic transmission and plasticity. These responses correlate with neurodegeneration as well as the development of neurodegenerative diseases that have age as a risk factor (Ahmed et al., 2020; Kapoor and Nation, 2021; Razani et al., 2021; Goyal et al., 2023; Jurcau et al., 2024; Wareham et al., 2024). Furthermore, shared events such as the increased prosurvival signaling and changes in regulation of metabolism and calcium dynamics suggest that alterations in both neuronal types could be interpreted as an attempt of the cells to reestablish homeostatic levels of cellular function and neuronal connectivity during aging (Toescu and Verkhratsky, 2007; Yang et al., 2023).

### Sex differences in age effects on gene expression

**Figure 5.**
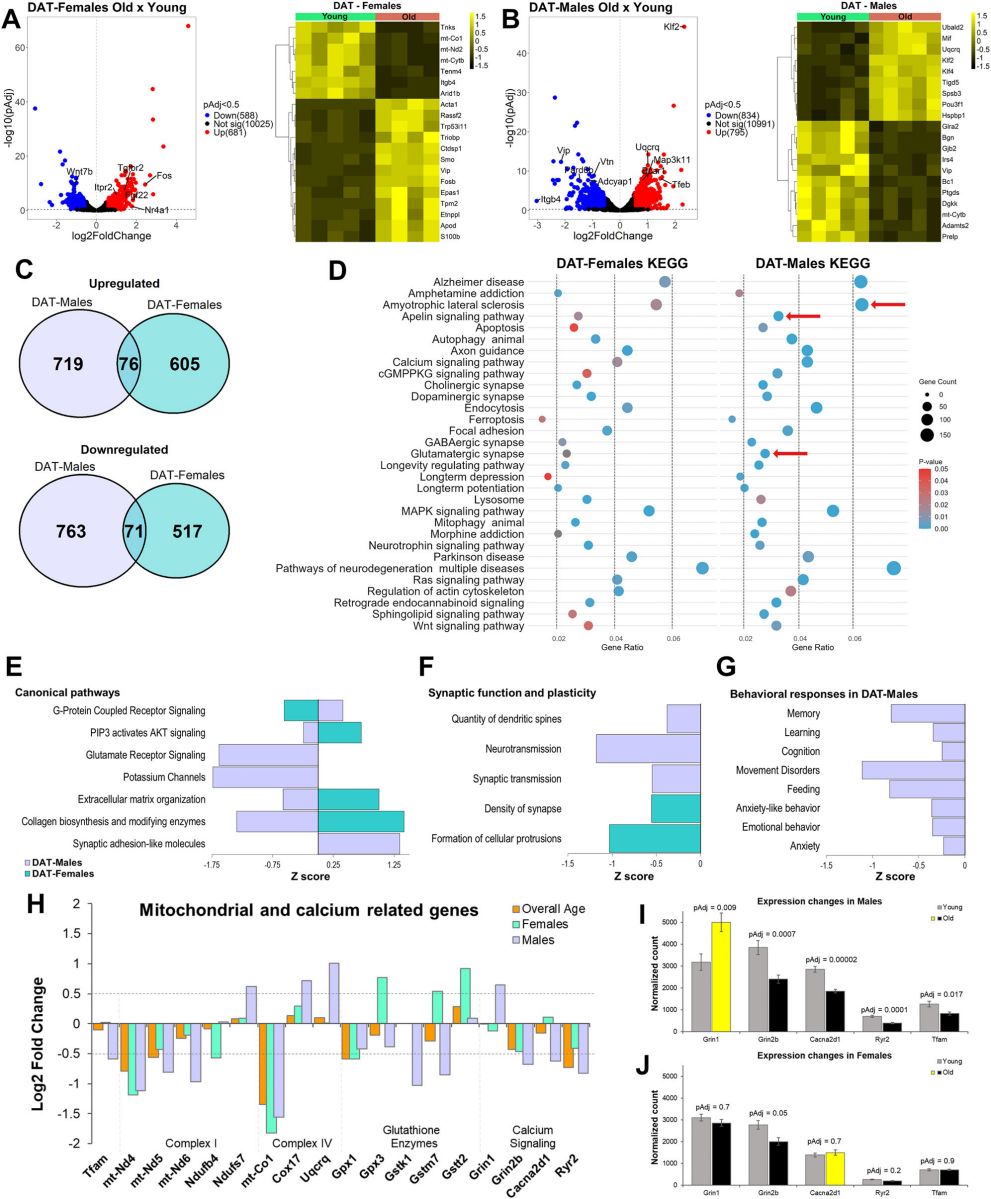
Sex-separate expression changes in dopamine neurons with age. ***A***, A volcano plot of differential expression analysis for females and heatmap of the top DEG (cutoff, Log2FC > |0.5|; adjusted *p* < 0.5). ***B***, A volcano plot of differential expression analysis for males and heatmap of top DEGs. ***C***, Intersected up- and downregulated genes with age, between males and females. ***D***, Overall KEGG pathway analysis showing the most represented terms in both groups. The use of the same scale allows for comparison between predicted changes in both females and males. Red arrows, Different changes in pathways. ***E***, IPA shows comparison of activated and deactivated paths for canonical pathways of both groups. ***F***, IPA analysis show comparison of the downregulated pathways involved in synaptic function and plasticity for both groups. ***G***, IPA analysis shows predicted behavioral pathways downregulated for males, which were not altered in females. Pathways analysis cutoff: *p* < 0.05 and *q* < 0.02. ***H***, Expression changes with age of mitochondrial-related and calcium signaling-related genes for overall aged (DAT;NuTRAP), males DAT;NuTRAP and females DAT;NuTRAP. Dotted lines show cutoff values for gene expression changes (Log2FC > |0.5|). ***I***, Expression changes of specific genes in males, showing increased (yellow) or decreased (black) expression with age and expression changes of the same genes in females (***J***). The adjusted *p* value (*p*_Adj_) is represented for each gene (significant changes were defined as having *p*_Adj_ < 0.5).

We next investigated whether age-related alterations in dopamine neurons from DAT;NuTRAP mice were differentially influenced by sex. Differential expression analysis in DAT-females showed 681 upregulated and 588 downregulated genes (Fig. 5*A*; GSE295369) that in general were consistently changed across all aged samples (Fig. 5*A*). In DAT-males, there were 795 genes upregulated and 834 downregulated (Fig. 5*B*; GSE295369), also consistent across samples (Fig. 5*B*). Out of 1,400 potentially upregulated genes (males + females), only 76 genes were shared between the two groups (Fig. 5*C*). A similar trend was observed for downregulated genes, where there was an overlap of 71 out of 1,351 genes (males + females; Fig. 5*C*). This result points toward substantial differences in dopamine neuron aging between sexes. Of the 1,269 DEGs identified in females, only 27 were present on the X chromosome, while in males 48 of the total 1,629 DEGs were located on the X chromosome. No Y-linked DEGs were observed in either dataset. Taken together, these results suggest that the large majority of the differences in gene expression between DAT-males and DAT-females are present on autosomal genes and are not due to sexual chromosome expression differences.

Despite the apparently large differences in age-driven gene expression changes for males and females, KEGG analysis performed on all DEGs in each group (Fig. 5*D*) revealed overlapping biological processes affected by aging, with similar gene ratios and *p* values. The top affected pathway in both groups was for genes connected with neurodegenerative diseases. There were, however, noteworthy differences in the pathway analysis, particularly in males. There was a higher difference in the expression of genes associated with amyotrophic lateral sclerosis (ALS), a disease known to be connected with age (Niccoli et al., 2017; Mehta et al., 2018) and is more prevalent in men (Zamani et al., 2024). The apelin pathway was also significantly enriched in males (*p* = 5.21 × 10^−5^) and was one of the most downregulated pathways in the overall DAT;NuTRAP analysis (Fig. 3*C*). As mentioned above, apelin signaling is affected by aging (Sauvant et al., 2014), is connected with several neurological disorders (Cheng et al., 2012), and is neuroprotective in mouse models of PD (Haghparast et al., 2018; Zhu et al., 2020).

Genes associated with glutamatergic signaling were apparently more altered in dopamine neurons from males than females (Fig. 5*D*; GSE295369). DAT-males exhibited downregulation of several ionotropic glutamate receptors (*Grin2b*, *Grid2*, and *Grik2*), suggesting a potential decrease of postsynaptic glutamatergic signaling. In contrast, DAT-males showed a significant increase in *Grin1* (Fig. 5*I*; GSE295369), which could indicate a compensation for overall changes in glutamate signaling. Finally, DAT-males displayed downregulation of metabotropic glutamate receptors *Grm7* and *Grm8*, which, in combination with the changes in expression of Gβƴ subunits (down, *Gng10* and *Gng11*; up, *Gng13*), suggest alterations of regulation of neurotransmitter release in presynaptic terminals (Castillo-Vazquez et al., 2024). We detected that transcript for the vesicular glutamate transporter Vglut2 (*Slc17a6*), a marker of glutamate cotransmission, was downregulated in both sexes but more pronounced in males (Log2FC = −1.1 vs −0.7; GSE295369). Vglut2 is thought to contribute to dopamine neuron resilience to aging and in Parkinson's models and is observed at higher levels in females than in males in flies, rats, and humans (Buck et al., 2021a,b). Decreased Vglut2 mRNA has been previously reported with aging in mouse dopamine neurons, although this may not result in altered protein expression in striatal terminals (Buck et al., 2025). Overall, our results involving alterations in glutamatergic signaling in DAT-males suggest a downregulation of both pre- and postsynaptic processes, which could correlate with age-related deficits in cognitive and behavioral responses (Castillo-Vazquez et al., 2024), and were not as prominent in DAT-females (GSE295369).

Ingenuity Pathway Analysis (IPA) of predicted activated or deactivated pathways (*p* < 0.05; *p*_Adj_ < 0.5) also revealed at least four canonical pathways with opposite effects between males and females (Fig. 5*E*; GSE295369). The analysis predicted a male-biased downregulation of glutamate receptors (as anticipated by KEGG analysis; Fig. 5*D*; GSE295369) and potassium channels. *Z*-score results also revealed that synaptic function and plasticity appear to be downregulated in both males and females (Fig. 5*F*; GSE295369), but the specific mechanisms involved may be different between the two groups. While in males there was a predicted downregulation of quantity of dendritic spines and neuronal/synaptic transmission, changes in females were associated with decreased synaptic density. Lastly, aged males demonstrated changes in gene expression connected with the downregulation of several behavioral responses (Fig. 5*G*; GSE295369), most of which have been associated with dopamine and may decline in aging individuals (Charles and Carstensen, 2010; Noda et al., 2020). This includes behavioral responses associated with movement disorders, calling to mind the hallmark symptoms of PD (Jankovic, 2008; Opara et al., 2017). Although we observed changes in expression of specific genes implicated in behavioral responses such as *Grin2b* (Fig. 5*I*,*J*) in females, we found no prediction of significant changes of these pathways in DAT-females (GSE295369).

One final difference of note between male and female DAT;NuTRAP mice involves mitochondrial dysfunction and calcium signaling (Fig. 5*H*–*J*). While all three datasets (overall aged, aged males, and aged females) showed mitochondrial dysfunction and alterations in calcium signaling as an effect of age, the changes were most prominent in males. Specifically, males showed a significant decreased in the expression of *Tfam*, the protein responsible for stabilizing and transcribing mitochondrial DNA (Ekstrand, 2004) and whose knock-out in dopamine neurons drives parkinsonian phenotype due to alterations in mitochondrial respiratory chain (Ekstrand et al., 2007; Beckstead and Howell, 2021). Downstream of *Tfam* regulation, we observed that several mitochondrial genes involved in complex I (Fig. 5*H*) were downregulated, and although the changes were observed in all three datasets, it was more accentuated in males. Deficiencies in mitochondrial complex I in dopamine neurons of the SNc are commonly seen in PD patients and animal models (Schapira et al., 1990; Swerdlow et al., 1996; González-Rodríguez et al., 2021). Interestingly, we also observed in males an increased expression of genes involved in complex IV (Fig. 5*H*), which could be interpreted as an attempt to provide a compensatory effect to the deficiency in complex I. Male mice have been reported to maintain an overall homeostasis of synaptic mitochondrial function, despite alterations in mitochondrial proteins and bioenergetics (Stauch et al., 2014). Furthermore, males displayed downregulation of several genes involved in glutathione metabolism while females surprisingly showed upregulation of the same genes (Fig. 5*H*). Glutathione is an intracellular antioxidant, with important protective function in dopaminergic neurons (Zeevalk et al., 1997), and its decrease is also observed in PD patients (Perry et al., 1982). These differences in glutathione metabolism genes suggest that female dopamine neurons likely have better mechanisms to handle oxidative stress than in males. In combination, these results suggest that dopamine neurons of aged males are more susceptible to mitochondrial dysfunction than in aged females, due to differences in several genes. Because mitochondria are critical to the homeostatic regulation of Ca^2+^ in dopamine neurons (Duchen, 2000; Surmeier et al., 2011), we looked further into the effects of age and observed more alterations in genes involved in calcium signaling in males than in females (Fig. 5*H*–*J*). This correlates with previously reported observations that age-driven alteration of calcium dynamics are more relevant in males (Branch et al., 2014; Howell et al., 2020), and altogether these data suggest that age-related calcium dynamics are connected to mitochondrial dysfunction and decreased ability of calcium handling in dopaminergic neurons in aged males.

Overall, these results identify sexual divergences in dopamine neuron gene expression with age that correlate with the prevalence of certain neurodegenerative diseases, particularly PD. Given the potential link to neurodegenerative processes, this information could be used to drive specific hypotheses involving the role of whole brain aging and its involvement in age-related neurodegeneration. Furthermore, sex biases should be taken into consideration while studying any age effect on dopaminergic circuits, behavioral processes, and related diseases.

### Sex differences in gene expression changes in GABA neurons

**Figure 6.**
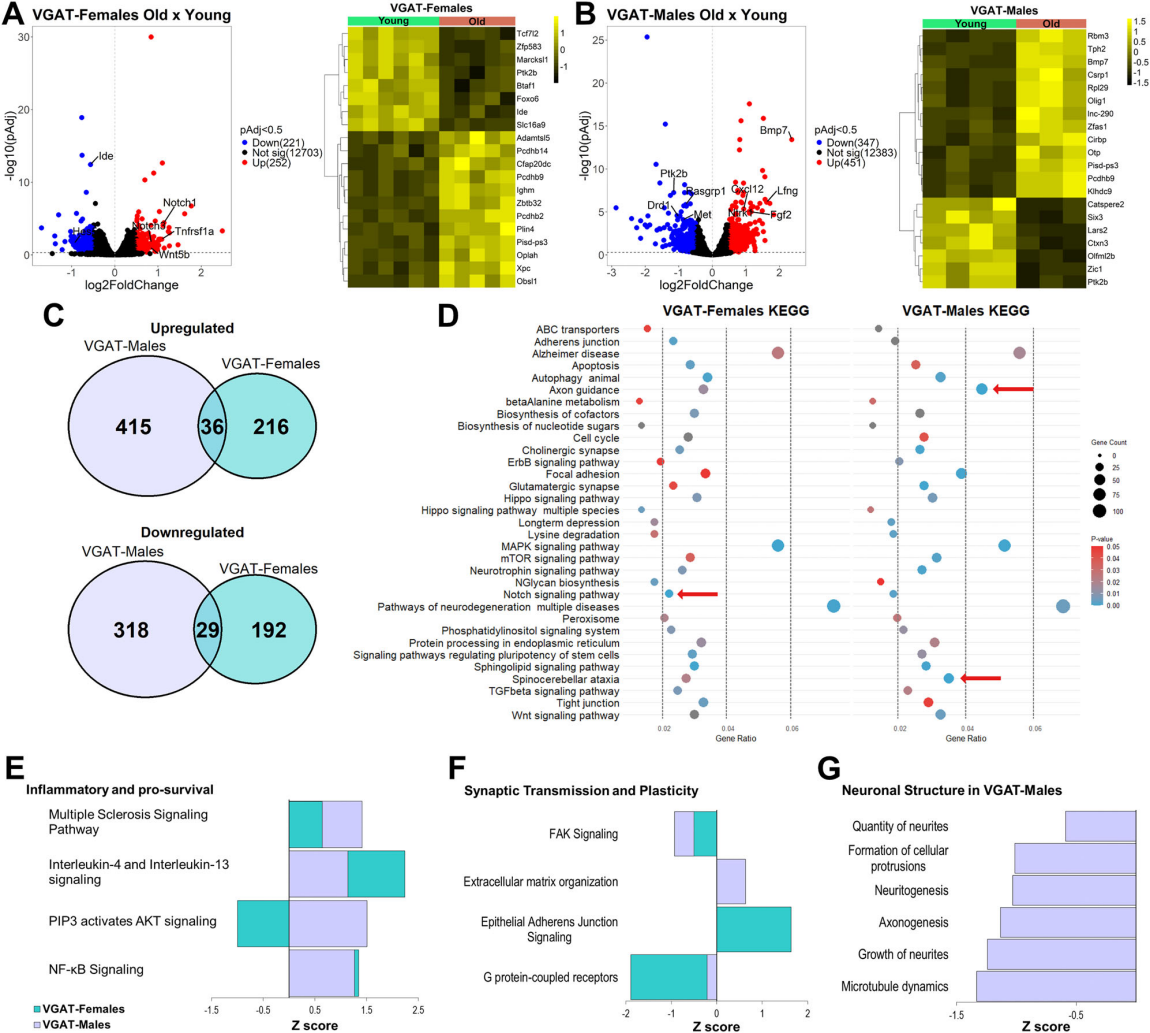
Sex-separate expression changes in GABA neurons with age. ***A***, A volcano plot of differential expression analysis for females and heatmap of the top DEG (cutoff, Log2FC > |0.5|; adjusted *p* < 0.5). ***B***, A volcano plot of differential expression analysis for males and heatmap of the top DEGs. ***C***, Intersected up- and downregulated genes with age, between males and females. ***D***, Overall KEGG pathway analysis showing the most represented terms in both groups. Red arrows, Different changes in pathways. ***E***, IPA shows comparison of activated and deactivated paths for inflammatory and prosurvival signaling in both groups. ***F***, IPA analysis shows comparison of the downregulated pathways involved in synaptic transmission and plasticity for both groups. ***G***, IPA analysis shows predicted neuronal structure pathways downregulated for males, which were not altered in females. Pathways analysis cutoff: *p* < 0.05 and *q* < 0.02.

The last step in our analysis was to investigate if there were sex-related differences in aged GABA neurons using VGAT;NuTRAP mice. Differential expression analysis in old females revealed upregulation of 252 and downregulation of 221 genes (GSE295369) and that the top DEGs were consistently changed across samples (Fig. 6*A*). In males, we observed 451 upregulated and 347 downregulated genes (GSE295369), with the top DEGs again changing consistently across samples (Fig. 6*B*). Of these, females and males shared just 36 common upregulated genes (Fig. 6*C*) and 29 common downregulated genes, again suggesting prominent sex effects at the level of individual DEGs. Out of all age-related DEGs in VGAT-males, 33 were X-linked genes, while in VGAT-females 6 genes were X-linked genes, with an overlap of 4 common genes between both groups. This suggests that the majority of sex effects in gene expression were autosomal and not associated with sex chromosomes.

As observed with DAT;NuTRAP mice, KEGG pathways analysis of the VGAT groups (Fig. 6*D*) showed several overlapping pathways with similar gene ratios and *p* values. Also in agreement with DAT;NuTRAP data, the highest change in gene expression for both males and females was in genes connected to pathways of neurodegeneration (Fig. 6*D*), corroborating what is known about aging being a factor in neurodegenerative diseases (Baker and Petersen, 2018), but in a second type of midbrain neuron. Some pathways exhibited differences between the sexes; for example, males had considerably more altered genes related to spinocerebellar ataxia, similar to what was observed in dopamine neurons that had more ALS-related DEGs. Although there are no reports of higher male prevalence of spinocerebellar ataxia, spinocerebellar ataxia type 2 is clinically related to both PD and ALS (Elden et al., 2010; Antenora et al., 2017; Ferrari et al., 2024), which are more prevalent in male patients. Other sex-biased pathways included axon guidance in males and a higher gene ratio in females correlating with the Notch signaling pathway (Fig. 6*D*), which is connected with AD (Kapoor and Nation, 2021) and has been shown to be activated in AD mouse models (Chen et al., 2019).

Pathway analysis showed that a few prosurvival pathways are activated with age in GABA neurons, for both males and females (Fig. 6*E*; GSE295369). Curiously, one pathway that changed with age in opposite directions for males and females, the “PIP3 activates AKT signaling” pathway, is also an age-related sex–biased pathway in DAT;NuTRAP mice (Fig. 5*E*; GSE295369). PIP3/AKT signaling has a variety of functions in neurons and is connected to both prosurvival signaling (Hu et al., 2018) and synaptic plasticity (Jaworski et al., 2005; Kumar et al., 2005). Therefore, the exact effect of the activation or deactivation of PIP3/AKT in each cell type (dopamine or GABA neurons), or sex, with aging remains to be determined. IPA analysis also indicated that synaptic transmission and plasticity were affected in both males and females with age (Fig. 6*F*; GSE295369), although these changes appear to be mostly similar between the two sexes. One last observation was that males presented changes in a variety of processes predicted to be involved in neuronal structures (Fig. 6*G*; GSE295369), but these pathways were not significantly altered in VGAT-females (*p* < 0.05; *p*_Adj_ < 0.5).

These results suggest that while sexually divergent changes in gene expression occur with age in both dopamine and GABA neurons, they tend to converge into similar effects on biological processes. Overall, sex effects appeared to be more prominent in dopamine than in GABA neurons.

## Discussion

While brain aging inevitably produces some decline in cognitive and behavioral function, whether it leads to the development of neurodegenerative disease (Anderton, 1997; Wyss-Coray, 2016; Hou et al., 2019) depends strongly on the individual. Furthermore, while susceptibility to neurodegeneration and cell death can vary dramatically by cell type and brain region, the factors that are ultimately responsible for this variance are not well understood. Studies performed in humans and laboratory rodent models place midbrain dopamine neurons and their regulatory circuits at the crux of some of the most common age-driven brain diseases (Rollo, 2009; Nobili et al., 2017; Trist et al., 2019; Noda et al., 2020; Blankenship et al., 2024). GABA neurons in the same area may represent a more resilient neuron type, although inhibitory circuits do have suspected roles in brain disease (Błaszczyk, 2016; Purves-Tyson et al., 2021). Here we sought a better understanding of the effects of normal aging on two distinct neuron types in a single brain area by investigating the translatomics changes in midbrain dopamine and GABA neurons. To do so, we used genetic crosses to create and validate two neuron-type–specific NuTRAP lines (DAT;NuTRAP and VGAT;NuTRAP). GFP ribo-tags expressed only in cells containing *Cre-recombinase* allow for the acquisition of neuron-type–enriched RNA-seq datasets (Fig. 1). Of note, the NuTRAP construct also supported mCherry expression on the nuclei of Cre-positive neurons, which was not the subject of the current study but will allow for epigenomic studies in the future. This tagging approach also avoids the challenges (e.g., cellular fragility and lack of cell surface markers) of flow sorting neurons (Martin et al., 2017). Overall, the present findings illuminate some of the common and divergent pathways of age-driven molecular alterations in both neuronal types, offering new insights into the mechanisms underlying neuronal vulnerability and resilience in neurodegenerative contexts.

Dopamine neurons exhibited extensive transcriptional changes with aging (Fig. 3), including the upregulation of response to inflammation and prosurvival pathways (e.g., MAPK, PI3K/Akt, and Notch signaling) and the downregulation of synaptic and cell signaling pathways (e.g., calcium, cAMP, apelin signaling). The combination of up- and downregulation events reflect a global change in neuronal homeostasis, an effect common to aged neurons observed in other regions of the brain. Although several of the findings observed here are consistent with those reported previously (Toescu and Verkhratsky, 2007; Sauvant et al., 2014; Ahmed et al., 2020; Kapoor and Nation, 2021), their presence in dopamine neurons likely has broad consequences due to their regulatory role in a variety of functional processes. Moreover, we here and others (Kilfeather et al., 2024) observed aging alterations in mitochondrial gene expression, a known outcome (Amorim et al., 2022) that can prompt dopaminergic dysfunction and is often detected in patients and animal models of PD (Bose and Beal, 2016; Grimm and Eckert, 2017; Moradi Vastegani et al., 2023). Laboratory rodents do not naturally develop PD or AD, and yet, the overall molecular events detected in dopamine neurons during healthy aging provide an important link between age-related dysfunction and the age-dependent development of these neurodegenerative diseases (Haghparast et al., 2018; Angelopoulou et al., 2021; Bohush et al., 2021; Goyal et al., 2023). Notably, the downregulation of synaptic genes aligns with literature showing age-related epigenetic changes as key factors affecting synaptic structure and function during aging (Azpurua and Eaton, 2015).

When compared with dopamine neurons, GABAergic neurons showed fewer transcriptional changes, which may reflect to some extent their historically hypothesized resilience (Rissman et al., 2007). One noteworthy alteration observed with aging in GABA neurons is the upregulation of genes involved in the synthesis of serotonin, including upregulation of *Tph2*, which codes for the rate-limiting enzyme in the canonical synthesis pathway. *Tph2* expression has been reported in the VTA of male rats (Carkaci-Salli et al., 2011) and is detected in low levels in GABA-releasing neurons of the midbrain (Allen Brain Cell Atlas: https://portal.brain-map.org; Yao et al., 2023). GABA neurons also displayed increased expression of the gene *Cyp2d22*, which is involved in an alternative, noncanonical serotonin synthesis pathway (Singh et al., 2009; Cheng et al., 2013). Increased cytochrome P450 2D (CYP2D) expression has been previously reported in aged female rat brainstem, which could indicate enhanced serotonin signaling (Haduch et al., 2022). The identification of enhanced serotonin synthesis specifically in midbrain GABA neurons raises the intriguing possibility that GABA–serotonin cotransmission could develop through the lifespan during normal aging. Furthermore, mRNA obtained from VGAT;NuTRAP mice included the transcript *Slc18a2*, which codes for the Type 2 vesicular monoamine transporter VMAT2, indicating that midbrain GABA neurons may be able to package serotonin for release. Neurons that synthesize both GABA and serotonin have been found in the lamprey (Barreiro-Iglesias et al., 2009) and sparingly in the rat raphe (Stamp and Semba, 1995), but have not been widely reported in mammalian brain. Additionally, pancreatic beta cells are reported to corelease GABA, serotonin, and ATP from dense core vesicles (Braun et al., 2007). Thus, while the specific neurons subpopulations involved are not known, the data here are consistent with the development of GABA–serotonin cotransmission in the midbrain throughout the process of normal aging. Furthermore, although there is a consensus of the overall importance of the effects of serotonergic signaling in the aged brain, particularly in AD patients (Meltzer, 1998), the individual events that occur are controversial (Mcentee and Crook, 1991), likely due to the different responses observed across areas of the brain (Ciranna, 2006). Overall, it appears that with aging, there is a global decrease in 5-HT receptors and an increase in serotonin turnover (Mcentee and Crook, 1991), which is consistent with the increased synthesis of serotonin-related enzymes observed in GABA neurons (Fig. 3*H*). On the other hand, the increased expression of these enzymes (Tph2, Pla2g4e, and Cyp2d22) also correlates with the increase of serotonergic signaling in the presence of inflammation and cellular stress (Singh et al., 2009; Chen and Miller, 2012; Liu et al., 2022), another event we observed in aged GABA neurons (Fig. 3*F*,*G*). Whatever the driving factors, serotonergic alterations certainly influence the excitatory and inhibitory inputs regulated by GABA neurons and could contribute to age-related cognitive decline (Rissman and Mobley, 2011).

Concerning the similarities in the aging of GABA and dopamine neurons, both neuronal types demonstrated overlapping pathways associated with increased response to inflammation and declining of synaptic structure and plasticity. As chronic inflammation and cellular senescence have been identified as two hallmarks of aging (López-Otín et al., 2023), it might be expected that aged dopamine and GABA neurons would also express genes connected with cellular senescence. Changes in Notch, MAPK, cAMP, and cytokine signaling were observed in both cells (Figs. 3, 4) and are signatures of the senescence-associated secretory phenotype (Jurcau et al., 2024). In association with changes in calcium homeostasis and synaptic structure and plasticity, these alterations point toward the occurrence of neuronal “senescence” in both (postmitotic) cell types (Baker and Petersen, 2018; Jurcau et al., 2024). These shared changes highlight some of the potential mechanistic links between neuronal aging and the increased vulnerability of these cells, particularly dopamine neurons, to neurodegeneration. Furthermore, some common findings could reveal potential targets for prevention therapy that could modulate inflammation and/or reinforce synaptic plasticity pathways (Zotey et al., 2023; Dhyani et al., 2024; Pinho et al., 2024).

Despite the undeniable evidence connecting age-related gene expression changes to deficits in neuronal function and neurodegeneration, our data also highlighted several mechanisms of adaptation that allowed dopaminergic and GABA neurons to maintain homeostasis during healthy aging. In fact, to maintain proper function, neurons in the aging brain knowingly undergo several changes in metabolism and connectivity (Aron et al., 2022). One such change is likely the increase in PI3K/Akt signaling pathway we observed in DAT;NuTRAP mice (Fig. 3*C*), which activates prosurvival signaling cascades during stress (Hu et al., 2018; Razani et al., 2021). Mitochondrial function adaptation is another mechanism previously observed in aged neurons (Stauch et al., 2014) that we identified here in dopamine neurons, particularly in aged males that showed upregulation of genes involved in complex IV (Fig. 5*H*). Dopaminergic neurons in females showed increased upregulation of genes involved in protection against oxidative stress (Fig. 5*H*; Zeevalk et al., 1997) and are likely an adaptive mechanism that confer defense of these cells against mitochondrial dysfunction. Furthermore, changes in synaptic connectivity and plasticity could also represent adaptive mechanisms during healthy aging, since observations suggest that reduced synaptic gene expression predicts longer lifespan among healthy individuals (Zullo et al., 2019; Aron et al., 2022). Some of the changes in calcium dynamics and signaling (Figs. 3*C*,*K*,*L*, 5*H*–*J*) are likely compensatory mechanisms promoted by these neurons as an attempt to decrease the levels of circulating intracellular calcium, particularly in situations of mitochondrial dysfunction (Toescu and Verkhratsky, 2004, 2007).

Concerning sex differences in the age-related transcripts, we found that these were prominent in autosomally encoded genes and were observed in both dopamine and GABA neurons. As age-related changes in estrogen and estrogen receptor levels promote alterations in female brains that affect synaptic connectivity, neuronal function, and gene expression (Brinton et al., 2015; Boyle et al., 2021), the detection of sex differences was anticipated. In fact, sex differences in gene expression are often conserved across species (Wapeesittipan and Joshi, 2023) and several areas of the brain (Berchtold et al., 2008; Hong et al., 2024), particularly during aging. Although dopamine neurons of the SNc appear to have fewer sex differences in gene expression patterns than other regions of the brain (Fass et al., 2024), age-driven sex effects in dopaminergic function and physiology have been reported (Howell et al., 2020; Buck et al., 2021b; Troyano-Rodriguez et al., 2023). Some of these events were reflected as altered gene expression particularly in males, such as decreased glutamate receptor signaling, potassium channel expression (Fig. 5*E*; GSE295369), alterations in mitochondrial proteomics and bioenergetics (Fig. 5*H*), and alteration in calcium dynamics (Fig. 5*H*,*I*). Interestingly, both dopamine and GABA datasets presented sex differences that appeared to match up with the known prevalence of neurodegenerative diseases (Abbas et al., 2018; Mehta et al., 2018; Fass et al., 2024). Alterations in dopamine neurons connected to PD and ALS were also reflected in the increased of gene expression changes connected with spinocerebellar ataxia in GABA neurons (Figs. 5*D*, 6*D*), which could reflect similarities in aging and susceptibility to neurodegeneration in the two cell types. Furthermore, some of the female findings such as altered Notch signaling of GABA neurons (Fig. 6*D*) could reflect the connection of the pathway to AD prevalence in females (Chen et al., 2019; Kapoor and Nation, 2021; Fass et al., 2024). Curiously, although females undergo major brain-related hormonal and systemic changes with age (Brinton et al., 2015), the present work (Figs. 5*G*, 6*G*; GSE295369) and published data concerning sex effects on neuronal aging (Howell et al., 2020; Buck et al., 2021b) suggest that midbrain dopamine and GABA neurons in males are more sensitive to age-driven changes than females. Studies of the hippocampus and a few other regions detected that male brains have globally decreased anabolic and catabolic capacity and are therefore more susceptible to neurodegeneration (Berchtold et al., 2008). Hence, the sex differences we observed between dopamine and GABA neurons with aging warrant further investigation and should be considered when studying the effects of aging on the midbrain and related circuits.

Although this work characterizes the translatomic effects of aging in dopaminergic and GABAergic neurons, one caveat is that the quasi-bulk nature of the NuTRAP approach did not allow for a further understanding of specific dopaminergic subpopulations (Azcorra et al., 2023). Future studies could integrate spatial transcriptomics in the midbrain or single-nuclei analyses to further unravel the complexity of age-driven changes in dopamine and GABA neurons, as has been observed in other regions of the brain (Gonzalez-Velasco et al., 2020; Hahn et al., 2023; Kilfeather et al., 2024). Furthermore, Patch-sequencing of dopamine neurons was recently demonstrated in a mouse AD model (Blankenship et al., 2024), and a similar approach could be used here to study subpopulations of physiologically characterized dopamine neurons with aging. Proteomics and functional studies will also be necessary to validate the roles of identified pathways in neuronal aging and disease progression.

In summary, the present work reveals molecular underpinnings of neuronal aging, highlighting shared and distinct changes in dopamine and GABA neurons while providing insights into mechanisms that could link aging to neurodegeneration. These findings offer a framework for understanding how aging shapes dopamine and GABA neuron function and susceptibility to neurodegenerative diseases, emphasizing the potential of targeting age-associated pathways for therapeutic interventions.

## Synthesis

Reviewing Editor: Arianna Maffei, Stony Brook University Decisions are customarily a result of the Reviewing Editor and the peer reviewers coming together and discussing their recommendations until a consensus is reached. When revisions are invited, a fact-based synthesis statement explaining their decision and outlining what is needed to prepare a revision will be listed below. The following reviewer(s) agreed to reveal their identity: Susana Mingote.

The reviewers agree that the manuscript is well written and provides an important contribution to the field. Both reviewers suggested adding details to methods and results sections to help readers better understand methodology, analysis and interpretation of the results.

Additional clarifications are suggested to improve the take home message of the significance section and add information about how this study impacts the field. The detailed reviewers' comments are provided below.

Reviewer #1

This is a well-written manuscript that makes an important contribution to the aging field by comparing, for the first time, how aging alters gene expression in ventral midbrain dopaminergic and GABAergic neurons. The following comments primarily focus on areas where additional detail and clarification would enhance the clarity and impact of the manuscript:

1. Significance Statement

I recommend revising the Significance Statement to more explicitly highlight the age-induced differences between GABAergic and dopaminergic populations. Since a central aim of the study is to compare how aging affects these two distinct neuronal types, emphasizing both the commonalities and the cell-type-specific differences in gene expression would strengthen the impact and clarity of the statement.

2. Introduction

a) When citing work implicating VTA dopamine neurons in Alzheimer's disease, please clarify that these studies were conducted in mice. For example, revise the sentence to:

"Conversely, recent work in mice has selectively implicated VTA dopamine neurons in the development of AD (Blankenship et al., 2024; Nobili et al., 2017; Spoleti et al., 2024)."

b) In the final paragraph of the introduction, it would be helpful to briefly explain the TRAP technology, how it enables cell-type-specific transcriptomic analysis and its advantages to other techniques such as flow sorting neurons. This context would be particularly valuable for students and readers who may not be familiar with the technique.

c) Also in the final paragraph, the summary of the main findings could be more specific regarding the differences observed between dopaminergic and GABAergic neurons-similar to how this is presented in the abstract. Being more explicit here would help highlight the key findings and better orient the reader to the study's contributions.

3. Methods

A) A more detailed description of the NuTRAP mice is necessary, specifically it would be important to describe the promotor for the GFP expression.

B) For transparency, I would advise that authors to include a schematic/drawing showing the portion of midbrain that was collected for the transcriptomic analysis and describe what regions included in the midbrain portions contain GABAergic cells. Although Dopaminergic cells in the midbrain are restricted to the SNc and the VTA, the same is not true of the GABAergic neurons.

4. Results

A) In the subsection "NuTRAP crosses successfully provide translatomes enriched for midbrain cell types," it appears that the verification of GFP expression in a cell type-specific manner (in DAT;NuTRAP and VGAT;NuTRAP mice) was conducted only in young animals. The authors should provide additional information or justification to support the assumption that GFP expression remains comparably efficient in older mice. This is important to ensure that observed age-related transcriptional differences are not confounded by potential age-dependent differences in labeling efficiency.

B) In the same subsection, it would be very helpful if the authors more clearly defined the terms "input" and "positive fractions" in the Results section. For instance, an explanation could be added to the sentence: "Differential analysis of the RNA-seq data between positive and input fractions for all DAT;NuTRAP groups..." Providing clarification of this commonly used jargon would improve accessibility for a broader audience, particularly those less familiar with transcriptomic analysis.

C) The manuscript reports that several upregulated genes in aged GABAergic neurons are involved in serotonergic synaptic signaling, including Tph2, which encodes tryptophan hydroxylase-a key enzyme in serotonin synthesis. This raises the intriguing possibility that aging midbrain GABA neurons may begin to synthesize, and potentially co-release, serotonin. The authors should clarify this point in the Results section and further elaborate on the implications of this finding in the Discussion.

D) Regarding sex differences, the authors state: "Finally, genes associated with glutamatergic synapses were apparently more altered in males than in females (Figure 5D), an age-related sex effect that has been previously reported (Buck et al., 2021)." However, it is unclear which specific genes associated with glutamatergic synapses were altered, and whether they were upregulated or downregulated in males. The findings of Buck et al. (2021) highlight changes in VGLUT2 expression and glutamate co-transmission. It is not clear whether the current study observed changes in genes encoding glutamate receptors (which would affect dopaminergic postsynaptic responses to glutamatergic inputs) or in genes involved in glutamate packaging (which would affect dopamine neuron glutamate co-transmission). These represent distinct facets of glutamatergic function, and clarifying which pathways are affected is important for interpreting the relevance and implications of the reported sex-specific effects.

E) In the same section related to sex differences (page 14), the authors state: "Lastly, aged males demonstrated changes in gene expression connected with the downregulation of several behavioral responses (Figure 5G), most of which have been associated with dopamine and may decline in aging individuals (Charles &Carstensen, 2010; Noda et al., 2020)." It would be important to clarify whether any similar changes in these behavioral pathways were observed in females.

5. Discussion

On page 18, the authors state: "In particular, the increased expression of the gene Cyp2d22, which codifies one of the enzymes involved in the synthesis of serotonin, has been reported to be involved in an alternative serotonin synthesis pathway (Haduch et al., 2015)." This, along with the upregulation of Tph2, raises important questions about the functional implications of these findings. Do these changes suggest that GABAergic neurons in aged mice begin to synthesize and potentially release serotonin? Has GABA-serotonin co-transmission ever been reported in the midbrain before? Clarifying whether this gene expression shift translates into functional serotonergic signaling from GABA neurons would be a significant point for discussion.

6. Figures

a) In figure 1C and 1D, please include the age of the mice in which the experiment was done.

b) In the figure legend for panels 1E and 1F, the reference to the red-labeled dataset is missing.

Supplemental tables

The Extended Data primarily focuses on gene lists from the comparative analyses with Hahn et al. (2023) and Kilfeather et al. (2024). However, it would be highly valuable to include supplementary tables listing the differentially expressed genes (DEGs) within the key functional pathways highlighted in all the main figures. For example, genes related to behavioral responses-such as those associated with memory, learning, and cognition-are reported to be downregulated in dopaminergic neurons in males. Providing a table that clearly outlines these DEGs, organized by functional category and pathway, would significantly improve the transparency and utility of the dataset for readers and future studies.

Reviewer #2

To elucidate the impact of aging on midbrain dopamine and GABAergic neurons of mice, the authors employed a genetic, cell type-specific Translating Ribosome Affinity Purification approach to characterize gene expression changes across the lifespan in mice and to assess cellular alterations associated with healthy brain aging. They used sophisticated and well-validated approaches to analyze, depict and interpret their data. Their findings revealed differences in the regulation of individual genes between GABAergic and dopaminergic neurons of male and female mice, though both neuronal types were predicted to engage similar age-related biological pathways.

In male and female mice, the current study identified aging-related effects in both (DA, GABA) neuronal populations, up-regulation of inflammatory response and pro-survival signaling genes, as well as down-regulation of genes associated with synaptic transmission and plasticity. The authors concluded shared protective mechanisms that may support neuronal homeostasis and proper brain function during aging in rodents that may or may not relate to normal aging process in human or neurodegenerative diseases in human.

While the study provides descriptive insights into age-associated molecular changes in ventral midbrain dopamine and GABA neurons of male and female mice, a major limitation is the lack of clear correlation between gene regulation and downstream effects at the mRNA or protein level. This gap significantly limits the interpretability and translational relevance of the findings.

Furthermore, although the age of the mice is mentioned in the abstract, "young (6-10 months)" and "old (>21 months)", these age ranges are not clearly defined or justified in the methods or results sections. It is unclear why the authors used a broad age range for the young or older cohort and did not utilize more advanced-aged mice (e.g., 30 months old), which are available through the National Institute on Aging. A rationale for this choice should be provided.

Lastly, while the repeated references to Alzheimer's disease (AD) and Parkinson's disease (PD) in the context of aging are understandable, the authors must clearly state that wild-type rodents, unlike humans, do not naturally develop PD or AD. This is a well-known limitation of rodent models in neurodegeneration research and should be acknowledged explicitly.

## Author Response

RESPONSE TO REVIEWERS We are grateful to the reviewers for their insightful comments and suggestions. In response we have made several changes to the manuscript that address their concerns. We have rewritten sections of the Significance Statement, Introduction, and Results, added a new section on NuTRAP mice to the Materials and Methods, and added Discussion concerning the possibility of serotonin-GABA co-transmission. We have also added two new Tables (2 and S5) and updated schematics in Figure 1. Supplemental material is not allowed at eNeuro, but supplemental table are included here (Tables S1-S5) for double-blinded reviewing purposes only. These supplemental files, in addition to the original raw bam files, will be uploaded to the NCBI GEO repository upon acceptance.

Responses to specific critiques follow.

Reviewer 1:

1. Significance Statement R1. I recommend revising the Significance Statement to more explicitly highlight the age-induced differences between GABAergic and dopaminergic populations. Since a central aim of the study is to compare how aging affects these two distinct neuronal types, emphasizing both the commonalities and the cell-type-specific differences in gene expression would strengthen the impact and clarity of the statement.

A. We appreciate the suggestion. We have made modifications to the Significance Statement, adding specifics and highlighting commonalities and differences in findings between GABA and dopamine neurons.

2. Introduction R1. a) When citing work implicating VTA dopamine neurons in Alzheimer's disease, please clarify that these studies were conducted in mice. For example, revise the sentence to: "Conversely, recent work in mice has selectively implicated VTA dopamine neurons in the development of AD (Blankenship et al., 2024; Nobili et al., 2017; Spoleti et al., 2024)." A. We have now revised this and another similar sentence in the Discussion.

R1. b) In the final paragraph of the introduction, it would be helpful to briefly explain the TRAP technology, how it enables cell-type-specific transcriptomic analysis and its advantages to other techniques such as flow sorting neurons. This context would be particularly valuable for students and readers who may not be familiar with the technique.

A. Per this suggestion, we now explain how the use of NuTRAP-crosses allowed us to produce cell type-specific data, and explain the TRAP transgene in more detail. We also cite manuscripts that have applied the technique for similar purposes.

R1. c) Also in the final paragraph, the summary of the main findings could be more specific regarding the differences observed between dopaminergic and GABAergic neurons-similar to how this is presented in the abstract. Being more explicit here would help highlight the key findings and better orient the reader to the study's contributions.

A. Similar to the Significance Statement, we have now included a more defined summary of our findings in the last paragraph of the Introduction.

3. Methods R1. A) A more detailed description of the NuTRAP mice is necessary, specifically it would be important to describe the promotor for the GFP expression.

A. To address this, we have added a new section to the Materials and Methods titled "TRAP transgene and generation of NUTRAP-crosses." In it we provide more details about the TRAP construct, as well as the Cre-dependent expression of GFP-tagged ribosomal subunits.

R1. B) For transparency, I would advise that authors to include a schematic/drawing showing the portion of midbrain that was collected for the transcriptomic analysis and describe what regions included in the midbrain portions contain GABAergic cells. Although Dopaminergic cells in the midbrain are restricted to the SNc and the VTA, the same is not true of the GABAergic neurons.

A. We have added schematic drawings to Figure 1 of the area collected, which will clarify to the reader the specific regions of the brain we used as our samples.

4. Results R1. A) In the subsection "NuTRAP crosses successfully provide translatomes enriched for midbrain cell types," it appears that the verification of GFP expression in a cell type-specific manner (in DAT;NuTRAP and VGAT;NuTRAP mice) was conducted only in young animals. The authors should provide additional information or justification to support the assumption that GFP expression remains comparably efficient in older mice. This is important to ensure that observed age-related transcriptional differences are not confounded by potential age-dependent differences in labeling efficiency.

A. We apologize for the confusion; we did in fact stain both young and old mice. The images present in the Figures 1C and 1D are representative images. In this subsection we now describe GFP co-localization with DAT and GAD-67 antibodies. We have also changed the caption of Figure 1 to reflect that these are representative images. As previously demonstrated for RPE cells (PMID: 38723422) and microglia (PMID: 38058312), NuTRAP models provide equivalent enrichment across ages. In a similar manner to those authors, we performed analysis to demonstrate cell enrichment broken down by age groups (Figure 1E and 1F). We believe these two lines of data provide strong evidence that the isolation of GFP+ ribosome-bound mRNA is comparable between the age groups.

R1. B) In the same subsection, it would be very helpful if the authors more clearly defined the terms "input" and "positive fractions" in the Results section. For instance, an explanation could be added to the sentence: "Differential analysis of the RNA-seq data between positive and input fractions for all DAT;NuTRAP groups..." Providing clarification of this commonly used jargon would improve accessibility for a broader audience, particularly those less familiar with transcriptomic analysis.

A. We agree this would make the analysis more accessible to the reader. Early in the Results we now explain in better detail the terms "positive" and "input" fractions.

R1. C) The manuscript reports that several upregulated genes in aged GABAergic neurons are involved in serotonergic synaptic signaling, including Tph2, which encodes tryptophan hydroxylase-a key enzyme in serotonin synthesis. This raises the intriguing possibility that aging midbrain GABA neurons may begin to synthesize, and potentially co-release, serotonin. The authors should clarify this point in the Results section and further elaborate on the implications of this finding in the Discussion.

A. The increase in serotonergic synthesis genes in midbrain GABA neurons is intriguing, and we underemphasized this in the initial submission. Reports of potential GABA-serotonin co-transmission are sparse and have been confined to lamprey brain and a very small population of cells in the rat raphe. Information from the Allen Brain Cell Atlas seems to confirm expression of all three genes in GABA releasing neurons in the midbrain of adult mice. We have now made changes to our Results to emphasize the novelty of our findings, and developed this further in the Discussion as a (second, see below) example of midbrain neurons changing their neurotransmitter repertoire with age, including citations of published studies.

R1. D) Regarding sex differences, the authors state: "Finally, genes associated with glutamatergic synapses were apparently more altered in males than in females (Figure 5D), an age-related sex effect that has been previously reported (Buck et al., 2021)." However, it is unclear which specific genes associated with glutamatergic synapses were altered, and whether they were upregulated or downregulated in males. The findings of Buck et al. (2021) highlight changes in VGLUT2 expression and glutamate co-transmission. It is not clear whether the current study observed changes in genes encoding glutamate receptors (which would affect dopaminergic postsynaptic responses to glutamatergic inputs) or in genes involved in glutamate packaging (which would affect dopamine neuron glutamate co-transmission). These represent distinct facets of glutamatergic function, and clarifying which pathways are affected is important for interpreting the relevance and implications of the reported sex-specific effects.

A. We apologize for this oversight; given the role of Vglut2 expression with dopamine neurons and aging we should have provided more details. We have now edited this section of the Results by reporting specific findings in gene expression changes, and linking the text to Figure 5 and Table S5. We have also tried to be clear, when possible, about which changes were observed only in males, or in both sexes. We believe the revisions increase clarity.

R1. E) In the same section related to sex differences (page 14), the authors state: "Lastly, aged males demonstrated changes in gene expression connected with the downregulation of several behavioral responses (Figure 5G), most of which have been associated with dopamine and may decline in aging individuals (Charles &Carstensen, 2010; Noda et al., 2020)." It would be important to clarify whether any similar changes in these behavioral pathways were observed in females.

A. To provide transparency, we have added a new Table (S7) reporting all the changes in pathways in both males and females, with each specific DEG changed in the pathway. In addition, we have also pointed out in this section that behavioral pathways were not significantly changes in females.

5. Discussion R1. On page 18, the authors state: "In particular, the increased expression of the gene Cyp2d22, which codifies one of the enzymes involved in the synthesis of serotonin, has been reported to be involved in an alternative serotonin synthesis pathway (Haduch et al., 2015)." This, along with the upregulation of Tph2, raises important questions about the functional implications of these findings. Do these changes suggest that GABAergic neurons in aged mice begin to synthesize and potentially release serotonin? Has GABA-serotonin co-transmission ever been reported in the midbrain before? Clarifying whether this gene expression shift translates into functional serotonergic signaling from GABA neurons would be a significant point for discussion.

A. As we describe above, GABA-serotonin co-transmission has not been widely reported. We now do a better job at describing the novelty and implications of these findings in both the Results and Discussion.

6. Figures R1. a) In figure 1C and 1D, please include the age of the mice in which the experiment was done.

R1. b) In the figure legend for panels 1E and 1F, the reference to the red-labeled dataset is missing.

A. We have made corrections to both captions. We have also made changes to the manuscript to clarify that the GFP staining was performed in both young and old animals, and that the images present in figures 1C and 1D are representative examples.

Supplemental tables R1. The Extended Data primarily focuses on gene lists from the comparative analyses with Hahn et al. (2023) and Kilfeather et al. (2024). However, it would be highly valuable to include supplementary tables listing the differentially expressed genes (DEGs) within the key functional pathways highlighted in all the main figures. For example, genes related to behavioral responses-such as those associated with memory, learning, and cognition-are reported to be downregulated in dopaminergic neurons in males. Providing a table that clearly outlines these DEGs, organized by functional category and pathway, would significantly improve the transparency and utility of the dataset for readers and future studies.

We thank the reviewer for this suggestion. We have now created a new table (Table S5) that associates DEGs to their respective pathways and describes its biotype. In addition, it states side-by-side the differential expression (Log2FC) and the statistical values (pvalue and pAdj) for each gene in each comparison. Some values are represented in "NA" (Not available) because the read counts for the specific gene in a given group was below 10, and therefore the data was removed from the dataset during low count removal cleaning.

Reviewer 2:

R2. While the study provides descriptive insights into age-associated molecular changes in ventral midbrain dopamine and GABA neurons of male and female mice, a major limitation is the lack of clear correlation between gene regulation and downstream effects at the mRNA or protein level. This gap significantly limits the interpretability and translational relevance of the findings.

A. We agree with the reviewer that our study contains limitations, one of them being the lack of direct correlation to protein expression. An advantage of TRAP is that by analyzing translating mRNAs, instead of total RNA, this more closely matches protein expression (PMID: 31358845) as these transcripts are being actively translated. We now note this limitation in the Discussion. Ultimately, we hope the analyses will help set the ground for future functional and proteomics studies in these neuronal populations, by us and others.

R2. Furthermore, although the age of the mice is mentioned in the abstract, "young (6-10 months)" and "old (>21 months)", these age ranges are not clearly defined or justified in the methods or results sections. It is unclear why the authors used a broad age range for the young or older cohort and did not utilize more advanced-aged mice (e.g., 30 months old), which are available through the National Institute on Aging. A rationale for this choice should be provided.

A. We apologize for the oversight. 17 out of 20 mice in the young group were aged 8-9 months, and all old mice were 21 months of age with the exception of one 24-month-old mouse. To increase transparency, we have added a table (Table 2) reporting the age of each animal used in the entire study.

While we have used mice from NIA in the past, they do not breed NuTRAP mice in their aged rodent colony or crosses with cell type specific Cres, thus we had to use mice entirely bred in-house for this study. This was also the reason for the slight variations in age, as we were at the mercy of litter sizes and sex distributions for determining the best possible dates for brain collection. Finally, while we could and have previously used older (~30 month) mice, we wanted to study normal, healthy aging and did not want to select for an exceptionally long-lived population due to a potential survival bias. We also find that NuTRAP mice >24 months develop a series of health issues such as dermatitis, eye lesions, limited mobility and loss of appetite. We have now added age justification statements in the "Animals" section of the Materials and Methods.

R2. Lastly, while the repeated references to Alzheimer's disease (AD) and Parkinson's disease (PD) in the context of aging are understandable, the authors must clearly state that wild-type rodents, unlike humans, do not naturally develop PD or AD. This is a well-known limitation of rodent models in neurodegeneration research and should be acknowledged explicitly.

A. We now mention explicitly that rodents do not naturally develop Parkinson's or Alzheimer's disease.
